# Bimetallic Plasmonic Nanozyme‐Based Microneedle for Synergistic Ferroptosis Therapy of Melanoma

**DOI:** 10.1002/advs.202504203

**Published:** 2025-05-19

**Authors:** Wei Duan, Keying Xu, Yue Gao, Sheng Huang, Xueqian Xia, Xiang Liu, Shuangxue Pan, Chunpeng Jiao, Weijian Cheng, Yong Guo, Jingwen Zhao, Jia‐Wei Shen

**Affiliations:** ^1^ Key Laboratory of Elemene Class Anti‐Cancer Chinese Medicines School of Pharmacy Hangzhou Normal University Hangzhou 311121 P. R. China; ^2^ State Key Laboratory of Silicon and Advanced Semiconductor Materials Zhejiang University Hangzhou 310027 P. R. China; ^3^ State Key Laboratory of Molecular Engineering of Polymers (Fudan University) Shanghai 200438 P. R. China; ^4^ School of Chemistry and Chemical Engineering Zhejiang Sci‐Tech University Hangzhou 310018 P. R. China; ^5^ College of Chemistry and Chemical Engineering China University of Petroleum (East China) Qingdao 266580 P. R. China

**Keywords:** β‐ELE, bimetallic plasmonic nanozyme, ferroptosis, melanoma treatment, microneedle

## Abstract

Melanoma is the most common malignant skin tumor, characterized by complexity, invasiveness, and heterogeneity. Conventional therapies often yield poor outcomes, posing significant clinical challenges. Here, a microneedle (MN) patch that integrates nanozyme and traditional Chinese medicine (TCM) for ferroptosis pathway‐dependent combined therapy of melanoma is designed. To amplify therapeutic activity, a novel Au@MoS_2_ bimetallic plasmonic nanozyme (BPNzyme) is prepared through a simple aqueous synthesis strategy involving a two‐step process. Owing to the synergy between heterostructures, this rationally designed BPNzyme exhibits significantly enhanced therapeutic characteristics, including near‐infrared (NIR) photothermal effect, peroxidase‐like activity, and glutathione peroxidase‐like property, which can effectively reshape the tumor microenvironment and disrupt the redox homeostasis. Under the combined action of the TCM β‐elemene (β‐ELE) and NIR light, further enhancement of oxidative damage, lipid peroxidation, and glutathione peroxidase 4 expression downregulation are observed for skin tumor cells, validating the synergistic amplification of ferroptosis. Moreover, the transdermal delivery of BPNzyme and β‐ELE using the soluble hyaluronic acid MN patch effectively achieves 99.8% tumor growth suppression without significant systemic toxicity in vivo. These findings highlight the potential of the rationally designed BPNzyme‐based MN system as a promising innovative strategy for non‐invasive, efficient, and safe combination therapy of melanoma.

## Graphical abstract

A microneedle (MN) patch that integrates novel bimetallic plasmonic nanozyme (BPNzyme) and traditional Chinese medicine β‐elemene (β‐ELE) has been rationally designed for ferroptosis pathway‐dependent combined therapy of melanoma.

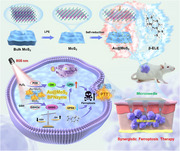

## 1 Introduction

Currently, skin cancer still remains one of the most challenging diseases of the skin system.^[^ ^1^ ^]^ The most common skin cancers in clinical practice include malignant cutaneous melanoma (CM), basal cell carcinoma (BCC) and squamous cell carcinoma (SCC).^[^ ^2^ ^]^ Of these, CM is the deadliest skin cancer, characterized by high aggressiveness and distal metastatic potential.^[^ ^3^ ^]^ Statistically, the 5‐year survival rate for patients with advanced CM (including lymph node and other metastases) is only 16%.^[^ ^4^ ^]^ The incidence of CM is predicted to increase by 50% and mortality is predicted to increase by 68% by 2040.^[^ ^5^ ^]^ Notably, the clinical efficacy of single surgery or chemotherapy is poor, and existing clinical treatments fail to significantly improve the overall survival rate of CM patients.^[^ ^6^ ^]^ Therefore, the development of efficient and safe novel treatments for malignant CM is urgent.

In recent years, microneedle (MN) has emerged as a transdermal drug delivery tool with advantages including painlessness, minimal invasiveness, and adjustable delivery dose.^[^ ^7^ ^]^ Consequently, it is expected to be an ideal option for localized treatment of melanoma. The dissoluble polymer MN system is capable of crossing the skin barrier above CM, thereby piercing the stratum corneum and creating microchannels.^[^ ^8^ ^]^ As the polymer dissolves, therapeutic molecules are deposited directly at the tumor site, enabling focused local drug delivery and release without passing through the internal circulation.^[^ ^9^ ^]^ To date, many soluble polymeric MN systems integrated with drug molecules, nanomaterials, photothermal agents, and photosensitizers have been designed for the treatment of CM.^[^ ^10^ ^]^ Despite the high delivery efficiencies displayed by these soluble MN systems, the therapeutic dose delivered to the tumor site is still insufficient owing to the limited area of MN patch. Furthermore, the impact of numerous therapeutic molecules is diminished by the complex and metabolically abnormal tumor microenvironment (TME).^[^ ^11^ ^]^ Considerable evidence indicates that tumors are metabolically aberrant diseases with abnormal redox homeostasis in the TME, as evidenced by high levels of reactive oxygen species (ROS) and the antioxidant glutathione (GSH).^[^ ^12^ ^]^ These properties promote tumor cell metabolism and proliferation, which ultimately severely compromising the anti‐tumor efficiency of therapeutic agents. For example, ferroptosis is a form of non‐apoptotic regulated cell death pathway and has been demonstrated as a significant way for cancer treatment. However, the antioxidant GSH, which is overexpressed in tumor cells, scavenges ROS and serves as a substrate for glutathione peroxidase 4 (GPx‐4) to catalyze the reduction of phospholipid hydroperoxides, thereby reducing the accumulation of lipid peroxides and protecting tumor cells from oxidative damage mediated by the ferroptosis pathway.^[^ ^13^ ^]^

To address the above issues, integration of ROS production and GSH consumption is required to remodel the redox state of the TME, thereby promoting the therapeutic efficacy of ferroptosis.^[^ ^14^ ^]^ Nanozymes are a class of nanomaterials with catalytic activities that mimic those of natural enzymes to modulate the TME in a variety of ways.^[^ ^15^ ^]^ For example, nanozymes with peroxidase (POD)‐like activity can induce the conversion of endogenous H_2_O_2_ in TME to highly toxic hydroxyl radicals (·OH), thus achieving tumor suppression by catalyzing the Fenton‐like reaction to cause oxidative damage of tumor cells.^[^ ^16^ ^]^ In addition, nanozymes with glutathione oxidase (GSHOx)‐like activity can deplete overexpressed GSH in TME and disrupt the antioxidant defense system, leading to the inactivation of GPx‐4 and accumulation of lipid peroxides (LPO) for boosting ferroptosis in tumor cells.^[^ ^17^ ^]^ The dual nanozyme‐triggered TME remodeling and redox homeostasis disruption can effectively enhance tumor ferroptosis and can further inhibit tumor progression in combination with other therapies. For example, our previous work has demonstrated that nanozymes with photothermal activity can synergistically enhance the therapeutic effect of CM on ferroptosis in combination with photothermal therapy (PTT).^[^ ^18^ ^]^ Generally, the design of ferroptosis‐inducing nanozyme systems should consider multiple aspects, such as simple preparation process, diverse catalytic activities, efficient catalytic efficiency, and multifunctionality, so that the ferroptosis effect can be amplified as much as possible for clinical applications. Hence, the rational design and construction of high‐performance nanozymes is still underway and play significant roles in the enhancement of ferroptosis pathway‐dependent tumor combination therapy.

Inspired by the cofactor stimulation mechanism of natural enzyme, heterogeneous metal hybridization has been considered as a practical and powerful approach to modulate the structure and properties of nanozymes.^[^ ^19^ ^]^ Compared to monometallic nanozyme, bimetallic nanozymes not only retain the unique properties of each metal component but also exhibit novel optical, electrical, and catalytic properties due to the synergistic interactions between the two metal nanostructures.^[^ ^20^ ^]^ The difference in electronegativity and redox potential of two counterparts in the bimetallic nanozyme leads to a synergistic interfacial interaction effect, which can affect electron transfer, substrate adsorption, and catalytic activity of nanozymes.^[^ ^21^ ^]^ Moreover, the localized surface plasmon resonance (LSPR) of noble metal nanoparticles can be attenuated by radiative pathways to produce localized thermal field enhancement near the interface of the bimetallic material, and hot electrons/holes can be produced by non‐radiative pathways to increase the photothermal conversion efficiency of metallic nanoparticles.^[^ ^22^ ^]^ For example, gold nanoparticles (Au NPs) with LSPR‐induced thermoelectric effect have been shown to significantly promote POD‐like catalytic activity and photothermal activity of CeO_2_ under near‐infrared (NIR) light irradiation.^[^ ^23^ ^]^ Accordingly, we believe that bimetallic plasmonic nanozyme (BPNzyme) developed based on the LSPR effect of noble metal nanoparticles can exhibit enhance multiple enzyme‐like and photothermal activities. However, the rational design of multifunctional BPNzyme by remodeling TME homeostasis for synergistically boosting ferroptosis remains to be explored. On the other hand, previous studies validate that remodeling of the TME by nanozymes can also increase the sensitivity of tumor cells to other therapeutic drugs, further boosting the tumor suppressive effect.^[^ ^24^ ^]^ Traditional Chinese medicine (TCM) has been used in clinical practice for thousands of years to prevent and treat disease.^[^ ^25^ ^]^ As a herbal extract used in TCM, β‐Elemene (β‐ELE) exhibits significant antitumor potential due to its favorable safety profile, high efficacy, and low production cost.^[^ ^26^ ^]^ Current clinical studies have demonstrated that β‐ELE serves as a promising adjuvant therapeutic agent, exhibiting synergistic effects in enhancing treatment efficacy against various malignancies.^[^ ^27^ ^]^ β‐ELE exerts anticancer effects through multiple pathways, including direct tumor cell elimination, apoptosis induction, reversal of multidrug resistance, and inhibition of metastasis.^[^ ^28^ ^]^ Notably, recent work revealed that β‐ELE's anticancer mechanism is closely linked to ferroptosis.^[^ ^29^ ^]^ Therefore, co‐delivery of BPNzyme and β‐ELE by MN is expected to be a promising strategy for synergistic activation to enhance ferroptosis in melanoma.

**Scheme 1:**
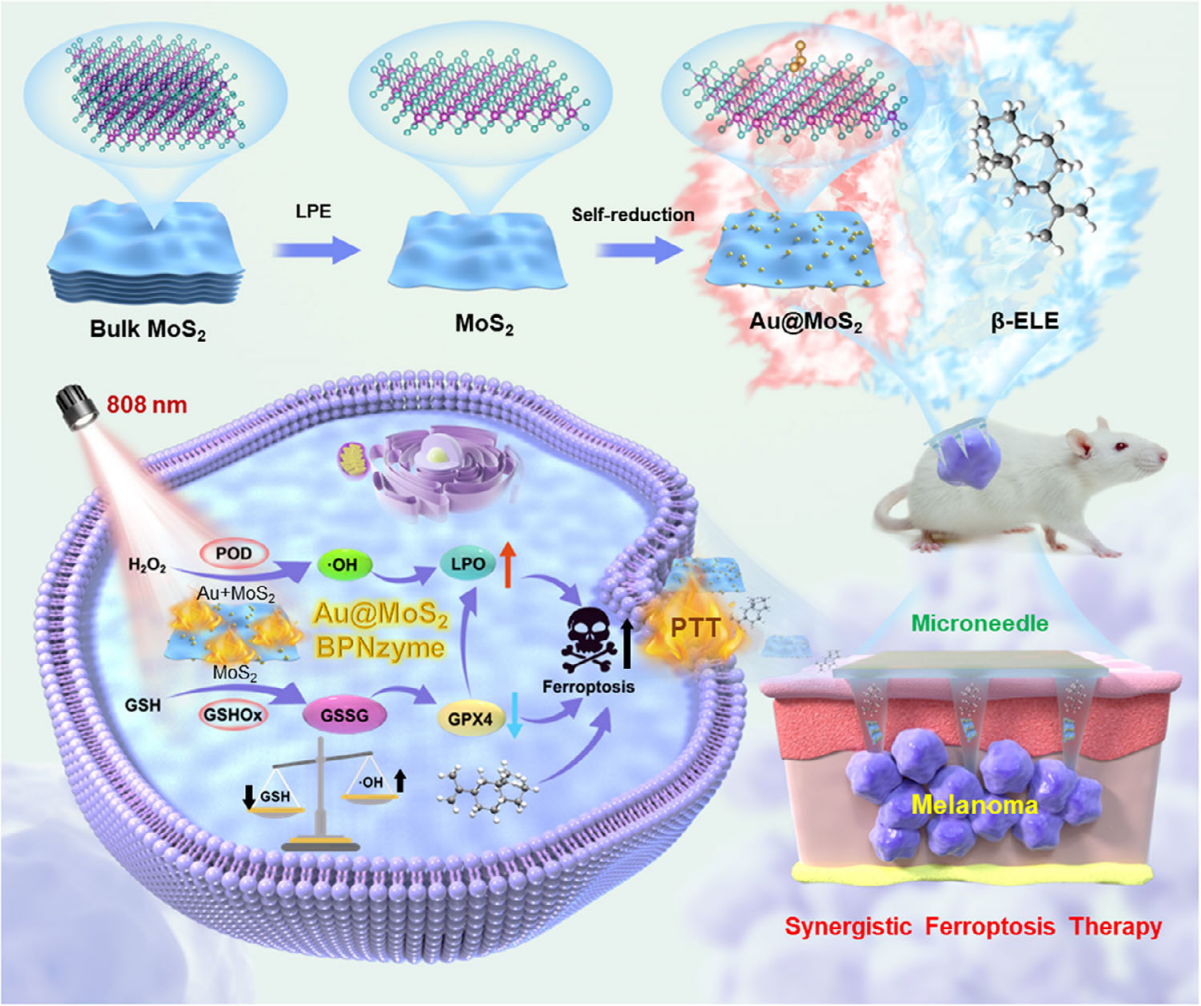
Schematic illustration of the fabrication process of Au@MoS_2_ and the transdermal delivery of Au@MoS_2_ and β‐ELE by MN for synergistically boosting ferroptosis‐mediated melanoma treatment.

On the basis of the above considerations, we have developed an innovative integrated MN system that synergistically activates the ferroptosis pathway for enhanced nanocatalytic/photothermal/TCM combination therapy (Scheme 1). This system leverages soluble MN for simultaneous transdermal delivery of a novel BPNzyme (Au modified MoS_2_, Au@MoS_2_) and the herbal molecule β‐ELE. Specifically, the 2D MoS_2_ nanoflakes were obtained from MoS_2_ powders by the classical liquid phase exfoliation (LPE) method. Subsequently, the intrinsic self‐reduction capability of MoS_2_ was harnessed to in situ grow Au NPs on MoS_2_ nanoflakes, resulting in the formation of Au@MoS_2_ BPNzyme. Finite‐difference time‐domain (FDTD) simulations and density functional theory (DFT) calculations confirmed that the NIR photothermal conversion and POD‐like nanocatalytic efficiencies of MoS_2_ are significantly enhanced by high‐density Au NPs doping, thereby maximizing its potential for combined photothermal/catalytic therapy. In vitro experiments demonstrated that dual‐active BPNzyme could exert efficient POD‐like activity to catalyze the decomposition of H_2_O_2_ to generate ·OH, while continuously depleting the overexpressed GSH through GSHOx‐like catalytic reaction. This dual action significantly reshaped the TME and further boost ferroptosis of tumor cells under the combined effect of TCM therapy and NIR photothermal treatment. To further enhance the therapeutic efficacy, BPNzyme and β‐ELE were integrated into biocompatible and water‐soluble hyaluronic acid (HA) MN for targeted local delivery to the melanoma lesion site. In vivo experiments proved that the prepared MN could effectively achieve combined nanocatalytic/photothermal/TCM treatment of melanoma with the highest tumor inhibition efficiency and negligible side effects. Taken together, our investigation not only provides valuable insights into the rational design of nanozyme, but also offers a novel and efficient combination strategy for boosting ferroptosis therapy of melanoma in clinical settings.

## 2 Results and Discussion

### 2.1 Preparation and Characterization of Au@MoS_2_

**Figure 1:**
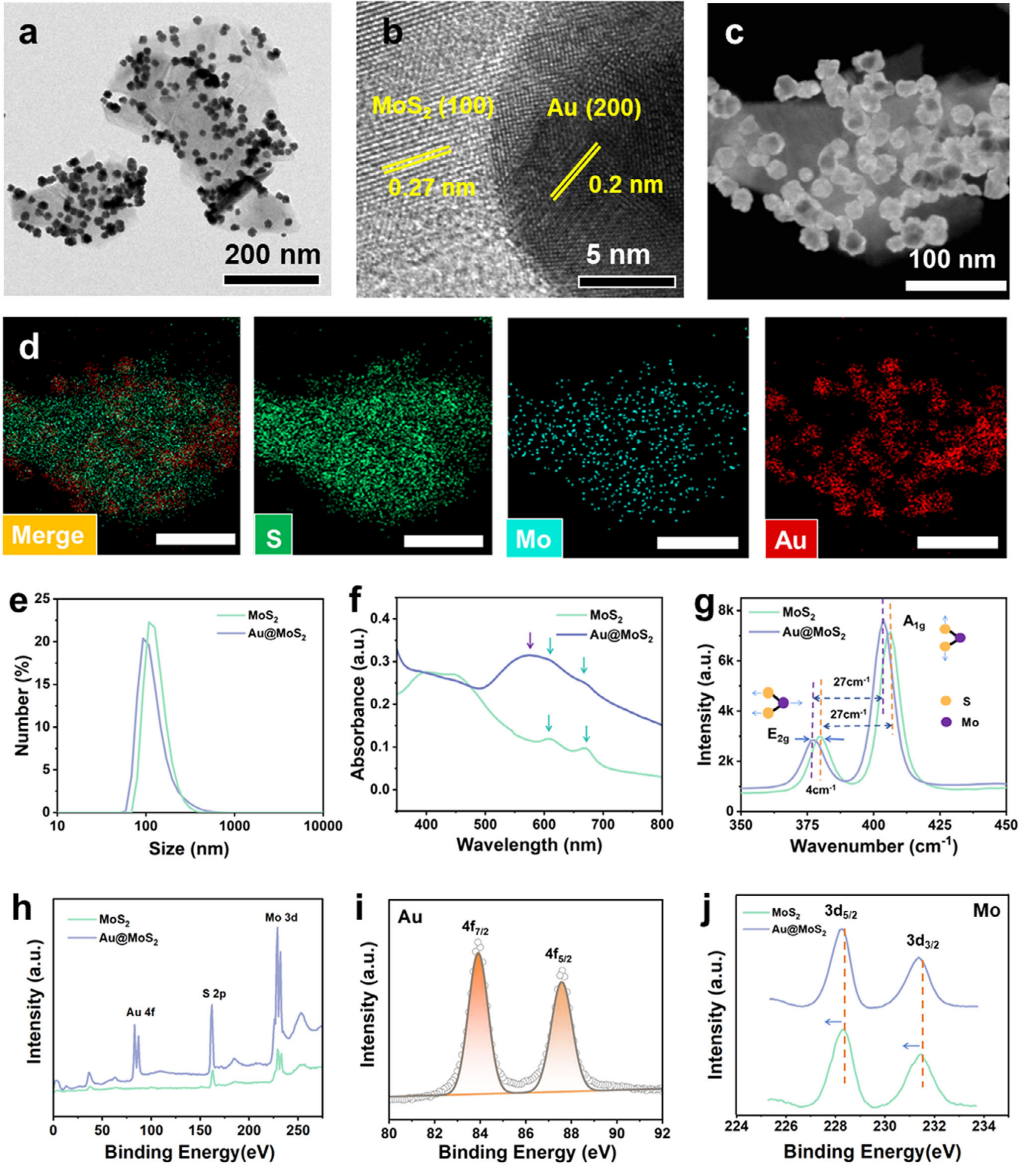
a) Typical TEM image of Au@MoS_2_. b) Typical high‐resolution TEM image of Au@MoS_2._ c) Typical HAADF‐STEM image of Au@MoS_2_. d) Corresponding EDX mapping images of Au@MoS_2_: S (green), Mo (blue), Au (red). The scale bars are 100 nm. e) Hydrodynamic size distribution of MoS_2_ and Au@MoS_2_. f) Typical UV–vis absorbance spectra of MoS_2_ and Au@MoS_2_. g) Typical Raman spectra of MoS_2_ and Au@MoS_2_. h) XPS survey of MoS_2_ and Au@MoS_2_. i) High‐resolution Au 4f XPS spectra of Au@MoS_2_. j) High‐resolution Mo 3d XPS spectra of MoS_2_ and Au@MoS_2_.

**Figure 2:**
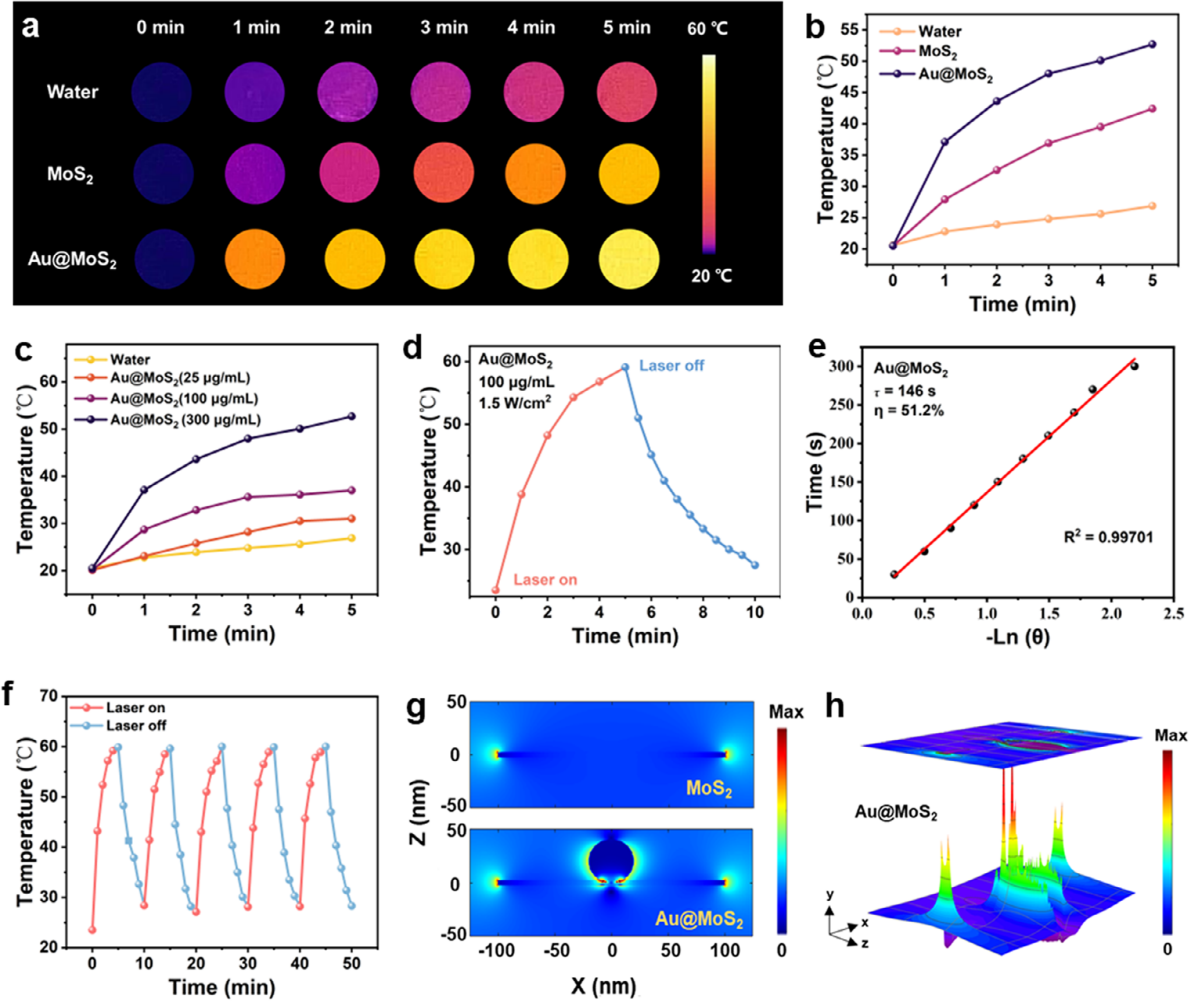
a) Thermal images and b) corresponding temperature change curves of water, MoS_2_, and Au@MoS_2_ under NIR laser radiation with power density of 1.0 W cm^−2^ for 5 min. c) Temperature change curves of the Au@MoS_2_ with different concentrations under NIR laser irradiation (808 nm, 1.0 W cm^−2^) for 5 min. d) Temperature change curve of Au@MoS_2_ (100 µg mL^−1^) with NIR irradiation (1.5 W cm^−2^) on and off. e) The photothermal conversion efficiency obtained from the relationship of linear time data versus −Ln θ. f) Photothermal stability tests of Au@MoS_2_ under NIR laser radiation with power density of 1.5 W cm^−2^ for 5 “on‐off” cycles. g) FDTD simulation results of electric field distribution for MoS_2_ and Au@MoS_2_, respectively. h) 3D Electric field distribution for Au@MoS_2_ under the 808 nm laser irradiation.

As shown in **Scheme** **1**, Au@MoS_2_ BPNzyme was produced by a two‐step method. The 2D MoS_2_ nanoflakes were first obtained by the classical polyvinylpyrrolidone (PVP)‐assisted LPE of MoS_2_ powders.^[^ ^30^ ^]^ Transmission electron microscopy (TEM) image exhibits β‐ that MoS_2_ nanoflakes has regular layered structure after stripping through LPE (Figure S1, Supporting Information). Atomic force microscopy (AFM) image shows that the thickness of the as‐prepared MoS_2_ nanosheets is ≈8 nm (Figure S2, Supporting Information). Subsequently, Au@MoS_2_ composites can be prepared by the self‐reduction reaction between MoS_2_ and HAuCl_4_ (redox pair: MoS_2_/AuCl_4_^‐^) using PVP as a stabilizer. During the reaction process, Au seeds can first form and disperse at the defects and edges of MoS_2_ nanoflakes, and then Au NPs can further grow along the Au seeds. Notably, the conventional synthesis of nanozymes typically involves harsh reaction conditions such as high temperature/pressure, toxic biochemical reagents, and additional reducing agents.^[^ ^31^ ^]^ In comparison, our synthetic method is greener and milder, proceeds in aqueous phase at room temperature without requiring additional reducing agents, demonstrating better cost‐effectiveness and greater potential for facilitating the clinical translation of nanozymes from laboratory to practical applications. As shown in **Figure** **1**a and 2D MoS_2_ nanoflakes are homogeneously deposited with a high density of Au NPs, which confirms the formation of heterogeneous nanostructure. In high‐resolution transmission electron microscopy (HRTEM) images, two lattice spacings of 0.2 nm and 0.27 nm were observed, corresponding to the (200) and (100) crystal planes of Au and MoS_2_, respectively (Figure 1b). Furthermore, high‐angle annular dark‐field scanning transmission electron microscopy (HAADF‐STEM) images (Figure 1c) and elemental mapping images (Figure 1d) prove the distribution of S, Mo and Au elements in the heterogeneous composite. As shown in Figure S3 (Supporting Information), the quantitative analysis by energy‐dispersive spectroscopy (EDS) revealed the following composition: 62.2 wt% Au, 23.4 wt% Mo, and 14.4 wt% S. The particle sizes of MoS_2_ and Au@MoS_2_ are measured by dynamic light scattering (DLS) to be 220 nm and 180 nm with polydispersity index (PDI) values of 0.212 and 0.238, respectively (Figure 1e). The size of Au@MoS_2_ is slightly smaller than that of MoS_2_ nanosheets, which may be due to the depletion of partial MoS_2_ by in situ reduction. During the synthesis of Au@MoS_2_, the defects and edges of MoS_2_, which contain unbound sulfur atoms, are continuously consumed in this process, and Au NPs are nucleated and deposited, leading to the reduction in the size of MoS_2_ nanosheets. The zeta potentials of Au@MoS_2_ and MoS_2_ are ≈−20 mV (Figure S4, Supporting Information), indicating that both nanomaterials are relatively stable in aqueous solution. Furthermore, the strorage stability of Au@MoS_2_ are tested in phosphate buffered saline (PBS) buffer solution during 12 days using DLS. As shown in Figure S5 (Supporting Information), the particle size of Au@MoS_2_ hardly changes significantly over 12 days at room temperature. The PDI values were all below 0.3, indicating good uniformity of the particle size distribution. The slight increase in particle size may be due to the mild aggregation of nanomaterials over time, which is common. The above results demonstrate that BPNzyme has good long‐term storage stability.

In addition, UV–vis spectroscopy shows that MoS_2_ exhibits characteristic absorption peaks at 400, 620, and 650 nm (Figure 1f).^[^ ^30^, ^32^ ^]^ Upon incorporation of Au NPs, an absorption peak at ≈550 nm is observed for the Au NPs with LSPR effect, indicating the successful synthesis of Au@MoS_2_ BPNzyme.^[^ ^33^ ^]^ Specifically, the introduction of a significant absorption peak near 550 nm in Au@MoS_2_, along with the weakening of the original absorption peak in MoS_2_, can be explained from several perspectives. First, the LSPR effect of Au NPs generates a new absorption peak in the visible light region (500–600 nm).^[^ ^34^ ^]^ Second, the formation of a heterostructure between Au and MoS_2_ leads to electron transfer from MoS_2_ to Au, altering the band structure of MoS_2_ and weakening its absorption peak.^[^ ^35^ ^]^ Additionally, Au doping may introduce defect states in MoS_2_, further suppressing its intrinsic light absorption.^[^ ^36^ ^]^ These results suggest that the Au NPs have been effectively deposited on the MoS_2_ surface, with the characteristic surface plasmon resonance peak of Au contributing to the observed spectral shift. Raman spectra shows that both MoS_2_ and Au@MoS_2_ have two characteristic vibrational peaks (Figure 1g). The E₂_g_ and A₁_g_ peaks of Au@MoS_2_ are shifted to lower wavenumbers by ≈4 cm^−1^ compared to pure MoS_2_, which can be attributed to the change in curvature of the MoS_2_ shell layer induced by the doping of Au, which introduces lattice strain, demonstrating the heterogeneous structure of Au@MoS_2_.^[^ ^36^, ^37^ ^]^ The elemental composition of the nanomaterial is further investigated using X‐ray photoelectron spectroscopy (XPS). As shown in Figure 1h, the XPS full spectrum shows the presence of Mo, S, and Au elements in Au@MoS_2_ while no Au signal is found in MoS_2_, confirming the successful incorporation of Au NPs into the MoS_2_ matrix. The Au 4f peaks are located at 87.89 eV (4f_5/2_) and 83.99 eV (4f_7/2_), which are typical peaks for Au NPs (Figure 1i).^[^ ^38^ ^]^ Besides, the Mo 3d spectrum has two peaks at 229.87 and 233.04 eV, corresponding to 3d_5/2_ and 3d_3/2_, respectively (Figure S6a, Supporting Information).^[^ ^39^ ^]^ The S 2p peaks consist of two peaks at 162.70 and 163.88 eV (Figure S6b, Supporting Information), confirming the expected charge states of Mo^4+^ and S^2‐^ in the MoS_2_ layer.^[^ ^40^ ^]^ To further investigate the effect of Au doping on MoS₂, we compared the binding energies of Mo 3d in MoS₂ and Au@MoS₂. As shown in Figure 1j, after Au modification, the Mo 3d spectrum exhibits a ≈0.3 eV shift toward lower binding energy. This shift is attributed to charge transfer from Au to MoS₂ at the bimetallic interface, providing further evidence of strong electronic interactions between Au and MoS₂.^[^ ^41^ ^]^ As shown in Figure S7 (Supporting Information), valance band‐XPS analysis reveals valence band positions of 0.95 eV for MoS_2_ and 0.17 eV for Au@MoS_2_. The above results indicate that after Au doping, the valence band energy is blue‐shifted, which is benefical for lowering the potential barrier for electron transfer and catalytic process.^[^ ^42^ ^]^ The above results provide strong evidence for the successful preparation of the Au@MoS_2_ bimetallic heterogeneous nanomaterials.

### 2.2 Photothermal Performance Investigation of Au@MoS_2_

In previous studies, Au NPs and MoS_2_ have been used as efficient photothermal agents for tumor therapy.^[^ ^43^ ^]^ In the present work, the developed BPNzyme is expected to realize increased photothermal conversion efficiency through heterojunction formation. First, we compare the photothermal conversion ability of different materials under NIR laser (808 nm) irradiation. The 808 nm laser operates within the near‐infrared (NIR) spectral range (700–1100 nm), a region often termed the “optical window” for biological tissues due to its minimal scattering and absorption.^[^ ^44^ ^]^ These properties enable deeper tissue penetration compared to shorter wavelengths, such as 600 nm red light. Although 1064 nm lasers exhibit superior penetration depth, the 808 nm wavelength remains adequate for targeting superficial tissues while offering practical advantages, including widespread clinical availability and cost‐effectiveness.^[^ ^45^ ^]^ As shown in **Figure** **2**a,b, under the NIR laser irradiation, the temperature of aqueous solution increases by only 5 °C, whereas the temperatures of the MoS_2_ and Au@ MoS_2_ solutions increase to 42 and 53 °C, respectively. The above results indicate that although both MoS_2_ and Au@MoS_2_ have NIR photothermal properties, the photothermal performance of Au@MoS_2_ is better than that of MoS_2_ at the same concentration. Next, different concentrations of Au@MoS_2_ solutions are exposed to 808 nm laser and the temperature changes of the solutions with increasing irradiation time are recorded. As shown in Figure 2c, the temperature increases as the concentration of the Au@MoS_2_ solution increases, indicating that the photothermal effect is concentration dependent. Further, the temperature variations of Au@MoS_2_ solution under 808 nm laser irradiation at different power densities (0.5, 1.0, 1.5, and 2.0 W cm^−2^) are also investigated. As shown in Figure S8 (Supporting Information), the temperature of Au@MoS_2_ solution is as high as 56 °C when the laser power is 2.0 W cm^−2^, while the temperature of pure water under the same conditions is only slightly higher than room temperature, showing that the laser power is positively correlated with the heating rate of Au@MoS_2_. The τ of Au@ MoS_2_ is calculated to be 146 s and the photothermal conversion efficiency (η) is 51.2% using the temperature rise‐cooling curve (Figure 2d,e). In comparison, the corresponding τ for MoS_2_ is 135 s and the photothermal conversion efficiency η is 39.3% (Figure S9, Supporting Information). Besides, the photothermal stablity test of Au@MoS_2_ (Figure 2f) shows that even after five heating and cooling cycles, Au@MoS_2_ BPNzyme still retains excellent photothermal response, indicating that it has good photothermal stability. As shown in Table S1 (Supporting Information), the η of Au@MoS_2_ significantly outperforms other reported bimetallic nanomaterials, demonstrating its strong potential as a promising candidate for PTT owing to its excellent photothermal efficiency and stability.

According to previous reports, spherical Au NPs exhibit relatively low photothermal conversion efficiency in the NIR region compared to materials such as Au nanorods.^[^ ^46^ ^]^ However, Au NPs doping significantly enhances the photothermal conversion ability of MoS₂. This enhancement can primarily be attributed to the LSPR effect of Au at the heterointerface, which amplifies the local electromagnetic field of MoS₂.^[^ ^34^ ^]^ Specifically, the potential spectral overlap between the exciton resonance of MoS₂ and the LSPR band of Au (typically 520–600 nm) may facilitate the generation of additional electron–hole pairs, thereby significantly improving the light absorption efficiency of MoS₂. Furthermore, after absorbing light energy via LSPR, the Au NPs may transfer energy to adjacent MoS₂ through near‐field dipole‐dipole coupling, increasing its carrier concentration and enhancing its photothermal conversion ability.^[^ ^47^ ^]^ To further evaluate the optical property, the bandgap energy of MoS_2_ was calculated from the UV–vis diffuse reflectance spectroscopy (UV–vis DRS) (Figure S10, Supporting Information). Similar to previous work, few‐layer MoS_2_ exhibits an indirect bandgap of ≈1.12 eV.^[^ ^48^ ^]^ Moreover, the bandgap of Au@MoS_2_ was reduced to 0.97 eV, which is mainly due to the introduction of impurity energy levels by the doping of Au, which is conducive to electron transfer.^[^ ^48^ ^]^ In the meantime, the narrow band gap can effectively promote the rapid nonradiative recombination of carriers, convert photon energy into lattice vibration, and help to improve the photothermal conversion ability of materials.^[^ ^49^ ^]^

To further elucidate the reason for significant increase in photothermal conversion capacity of Au@MoS_2_ BPNzyme, FDTD simulation is used to demonstrate the electromagnetic (EM) field distribution and enhancement. Figure 2g shows the electric field profiles of MoS_2_ and Au@MoS_2_ irradiated with NIR light at 808 nm, where the nanosphere represents one Au NP and the thin layer at the bottom represents one MoS_2_ nanoflake. The results suggest that the spatial electric field at the MoS_2_ edge and near the interface formed by the heterojunction is significantly enhanced, indicating that Au@MoS_2_ BPNzyme can effectively enhance NIR light absorption and photothermal conversion. As shown in Figure 2h, the 3D simulation result provides a clearer visualization of the spatial electric field distribution for Au@MoS_2_. Under 808 nm laser irradiation, the deposition of Au NPs significantly enhances the local electromagnetic field around MoS_2_. This improved EM field facilitates more efficient charge‐hole separation within the heterojunction and promotes the generation of hot electrons, resulting in a further improvement in photothermal performance.^[^ ^50^ ^]^

### 2.3 Nanozyme Activity Investigation of Au@MoS_2_

**Figure 3:**
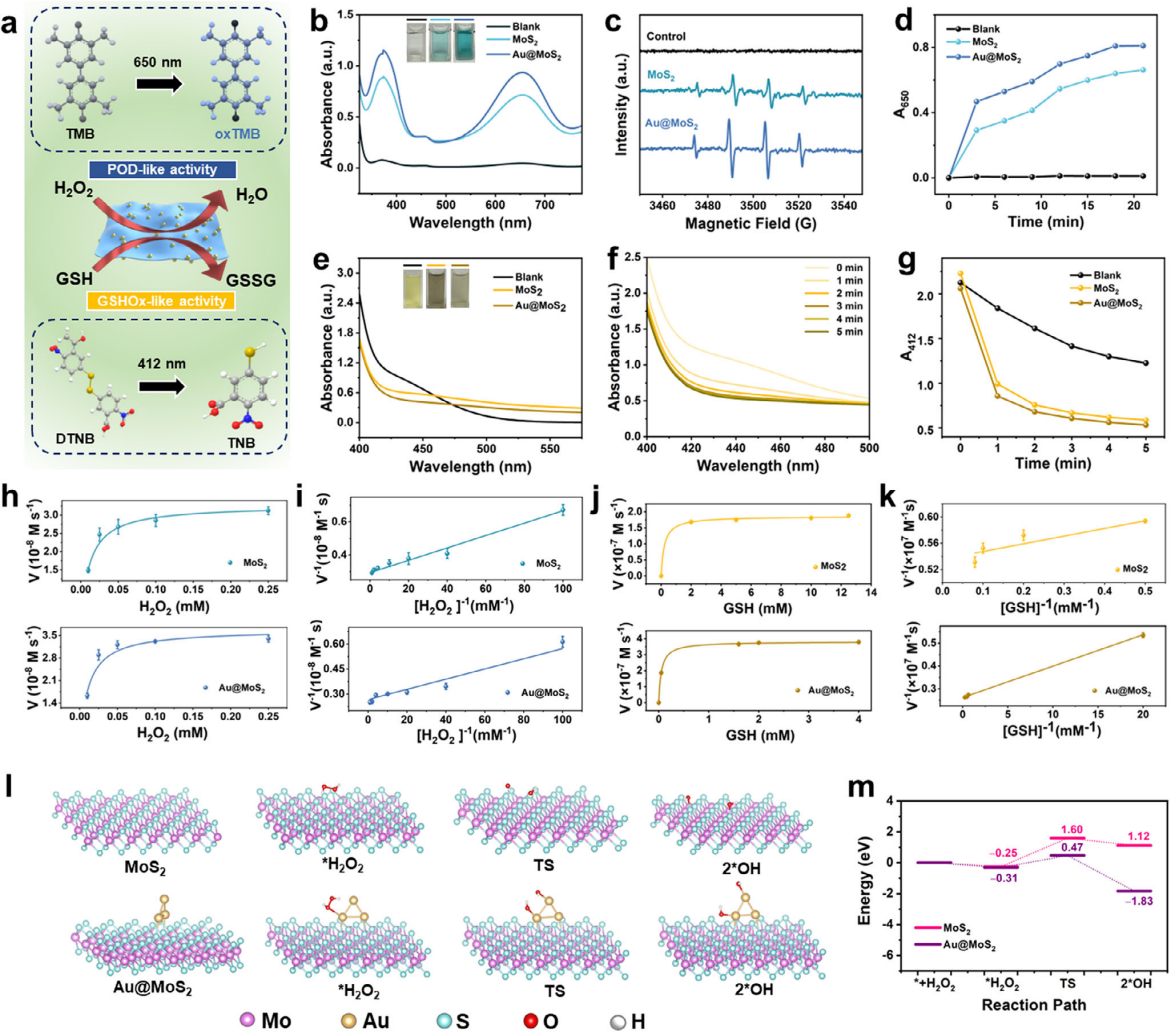
a) Schematic illustration of the POD‐/GSHOx‐like catalytic processes of Au@MoS_2_ nanozyme and their corresponding chromogenic substrate reaction. b) Typical UV–vis absorption spectra and photographs of TMB solution after catalysis by MoS_2_ and Au@MoS_2_. c) Typical EPR spectra of ·OH trapped by DMPO in the reaction solution after catalysis by MoS_2_ and Au@MoS_2_. d) Time‐dependent absorbance changes of TMB solution at 650 nm after catalysis by MoS_2_ and Au@MoS_2_ in 20 min. e) Typical UV–vis absorption spectra and photographs of DTNB solution after catalysis by MoS_2_ and Au@MoS_2_. f) Typical UV–vis absorption spectra of DTNB solution at 412 nm after catalysis by Au@MoS_2_ in 5 min. g) Time‐dependent absorbance change of DTNB solution at 412 nm after catalysis by MoS_2_ and Au@MoS_2_ in 5 min. h) Michaelis‐Menten kinetic curve for POD‐like MoS_2_ and Au@MoS_2_ with H_2_O_2_ as the substrate. i) Lineweaver‐Burk plot for MoS_2_ and Au@MoS_2_ with H_2_O_2_ as the substrate. j) Michaelis‐Menten kinetic curve for GSHOx‐like MoS_2_ and Au@MoS_2_ with GSH as the substrate. k) Lineweaver‐Burk plot for GSHOx‐like MoS_2_ and Au@MoS_2_ with GSH as the substrate. Data are presented as mean ± S.D. (*n*  = 3). l) Proposed POD‐like catalytic reaction mechanism of MoS_2_ and Au@MoS_2_ based on DFT calculation. (m) Free energy diagrams of MoS_2_ and Au@MoS_2_ in the catalytic process.

AuNPs and MoS_2_ have been widely demonstrated to have POD‐like activities that mimic natural horseradish peroxidase (HRP).^[^ ^51^ ^]^ More importantly, MoS_2_ has unsaturated sulfur atoms that can act as active sites for efficiently catalysis the oxidation of GSH.^[^ ^19a^ ^]^ Here, the dual nanozyme activities of Au@MoS_2_ BPNzyme are verified by two classical colorimetric reactions (**Figure** **3**a). The colorless substrate 3,3′,5,5′‐tetramethylbenzidine (TMB) can be oxidized by H_2_O_2_ under POD‐like catalysis to form a blue oxidized product (ox‐TMB) with a distinct absorption peak at 370 nm and 650 nm. The reaction of 5,5′‐dithiobis‐(2‐nitrobenzoic acid) (DTNB) and GSH can produce a yellow product with distinct absorbance peak at 412 nm. The oxidation of GSH catalyzed by GSHOx‐like nanozyme leads to a decrease in the absorbance value of the above reaction system at 412 nm, allowing DTNB to be used as a probe for the detection of GSH.

The POD‐like activity of different materials is first compared using the TMB colorimetric method. After introducing the same concentrations of MoS_2_ and Au@MoS_2_ into a mixture of H_2_O_2_ and TMB, the reaction was continued at a slightly acidic pH value of 5.5 at 37 °C for 5 min. The pH of TME is often lower than common tissues due to the Warburg effect, characterized by excessive glycolysis leading to substantial lactate production and accumulation within cells.^[^ ^52^ ^]^ As shown in Figure 3b, both MoS_2_ and Au@MoS_2_ groups show characteristic absorption peaks at 370 nm and 650 nm and the color of reaction solution is blue, indicating that TMB is successfully catalytically oxidized. Moreover, the absorbance value of the Au@MoS_2_ group is significantly higher than that of the MoS_2_ group. Further, electron paramagnetic resonance (EPR) spectroscopy was employed to confirm the generation of free radicals during the catalytic process. As shown in Figure 3c, using 5,5‐dimethyl‐1‐pyrroline‐N‐oxide (DMPO) as the radical scavenger, the EPR spectrum of Au@MoS₂ BPNzyme shows distinct 1:2:2:1 peak (characteristic of the·OH signal), which is not present in the blank control group. This result suggests that Au@MoS_2_ is more efficient in generating ·OH radicals compared to MoS_2_, providing further evidence for the enhanced POD‐like activity of Au@MoS_2_. The reaction system containing Au@MoS_2_ also shows the fastest rate of increase in absorbance values with reaction time among the three groups (Figure 3d). The absorbance values of different reaction systems stabilize after 10 min, indicating that the reaction has reached equilibrium by this time. In addition, the relative activities of Au@MoS_2_ are tested at different pH, temperature, and salt. Although it does not exhibit optimal activity at the pH corresponding to the TME (5.5‐6.5), Au@MoS_2_ retains relatively good POD‐like activity. (Figure S11a, Supporting Information). Meanwhile, Au@MoS_2_ exhibits optimal catalytic activity at 45–50 °C (Figure S11b, Supporting Information). Additionally, the inorganic salts present in the physiological environment could not significantly affect the mimic‐enzyme activity of Au@MoS_2_ (Figure S11c, Supporting Information).

Next, the GSHOx activity of Au@ MoS_2_ is analyzed using a thiol detection probe. The absorbance of the reaction system in the presence of MoS_2_ and Au@MoS_2_ decreases dramatically, indicating the effective GSHOx‐like activity of the MoS_2_ based material (Figure 3e). The absorbance at 412 nm of Au@MoS_2_ group decreases rapidly over time within 5 min (Figure 3f). As shown in Figure 3g, the change in absorbance at 412 nm for solution systems indicates that Au@MoS_2_ exhibits superior GSH depletion ability than MoS_2_. It is noteworthy that single Au NPs have been shown to lack the mimic‐GSHOx catalytic activity. The remarkable improvement in catalytic performance observed for Au@MoS_2_ can be attributed to the formation of bimetallic heterostructures, which facilitate the electron transfer process during the catalytic reaction. As a result, BPNzyme is expected to be more effective in increasing ROS levels and depleting cellular GSH, thereby significantly amplifying oxidative stress within tumor cells. As shown in Figure S12 (Supporting Information), Au@MoS_2_ maintains over 80% GSHOx‐like activity across a wide pH range, demonstrating excellent enzymatic stability. In addition, the NIR irradiation process can also effectively accelerate the absorbance changes in the TMB and DTNB chromogenic reactions, indicating that the NIR laser can enhance the catalytic reaction process of the nanozymes (Figure S13, Supporting Information).

To investigate the catalytic mechanism of dual enzyme activity, the steady‐state kinetics were analyzed using different concentrations of H_2_O_2_ and GSH as substrates, respectively. As shown in Figure 3h,i, according to the steady‐state kinetics using the typical Michaelis‐Menten equation, the Michaelis constant (*K*_m_) and the maximum reaction rate (*V*_max_) could be obtained. Compared with MoS_2_, Au@MoS_2_ has a lower *K*_m_ value (0.00907 mM) and a higher *V*_max_ value (3.533 × 10^−8^ M/s), indicating that Au@MoS_2_ has the better affinity and catalytic efficiency for the substrate. Therefore, Au@MoS_2_ can catalyze H_2_O_2_ decomposition more efficiently and produce more ·OH. Compared to MoS_2_, Au@MoS_2_ has similarly higher affinity for the substrate and catalytic efficiency when used as a mimic‐GSHOx (Figure 3j,k). The catalytic parameters of BPNzyme are compared with those of other nanozymes (Tables S2 and S3, Supporting Information). In terms of POD‐like and GSHOx‐like activities, the BPNzyme exhibit better *K*_m_ and *V*_max_ value, which is attributed to that the bimetallic interface formed by AuNPs and MoS_2_ can provide enhanced electron transfer for the enzyme‐like catalytic reaction, further accelerating the catalytic reaction kinetics.

In order to further elucidate the mechanism of enhanced catalytic performance of Au@MoS_2_, the POD‐like catalytic process of MoS_2_ and Au@MoS_2_ was investigated by DFT calculation. According to previous literature, the POD‐like catalytic process of metal nanozymes involves the decomposition of H_2_O_2_ into two ·OH on the catalyst surface.^[^ ^19^, ^53^ ^]^ Then, H_2_O_2_ can react efficiently on the MoS_2_ and Au@MoS_2_ surfaces to form unstable transition state intermediates, which can be rapidly destroyed to form ·OH (Figure 3l). As illustrated in Figure 3m and H₂O₂ initially adsorbs on the MoS_2_ surface with an adsorption energy of −0.25 eV, followed by the decomposition of H₂O₂, which requires overcoming a decomposition energy barrier of 1.85 eV, ultimately leading to the generation of two ·OH. This above process is endothermic (adsorption energy = 1.12 eV). In contrast, for the Au@MoS_2_ system, H₂O₂ preferentially adsorbs on the AuNPs surface, with an adsorption energy of −0.31 eV, slightly stronger than the adsorption of H₂O₂ on MoS_2_. The adsorbed H₂O₂ then decomposes into two ·OH, and the TS energy barrier calculated by DFT is 0.78 eV, significantly lower than the energy barrier for H₂O₂ decomposition on MoS_2_. This process is exothermic with an energy release of −1.83 eV. Therefore, compared with MoS_2_, Au@MoS_2_ is more favorable to catalyze the decomposition of H_2_O_2_ from both kinetic and thermodynamic perspectives, suggesting that the introduction of AuNPs plays a crucial role in promoting H_2_O_2_ decomposition. Based on the above experimental and theoretical calculations, convincing evidence is provided to support the excellent dual‐enzyme catalytic performance of our prepared BPNzyme, which is expected to reverse the TME through POD‐like activity and GSHOx‐like activity.

### 2.4 Evaluation of Anti‐Tumor Efficacy In Vitro

Inspired by the “compatibility theory” in Chinese medicine, we selected β‐ELE as a TCM model to further activate the ferroptosis pathway for combination therapy.^[^ ^54^ ^]^ β‐ELE is a representative active ingredient in TCM and a clinically approved anticancer drug that has shown exciting potential in the antitumor field due to its good safety profile, high efficacy, and low production cost.^[^ ^54b^ ^]^ Recently, Xie group discovers that the anticancer effect of β‐ELE is closely related to ferroptosis.^[^ ^29b^ ^]^ Benefiting from the excellent photothermal activity, POD‐like activity, and GSHOx‐like activity, the rationally designed Au@MoS_2_ BPNzyme is promising to efficiently regulate the TME and synergistically enhance ferroptosis in combination with β‐ELE for effective antitumor treatment.

**Figure 4:**
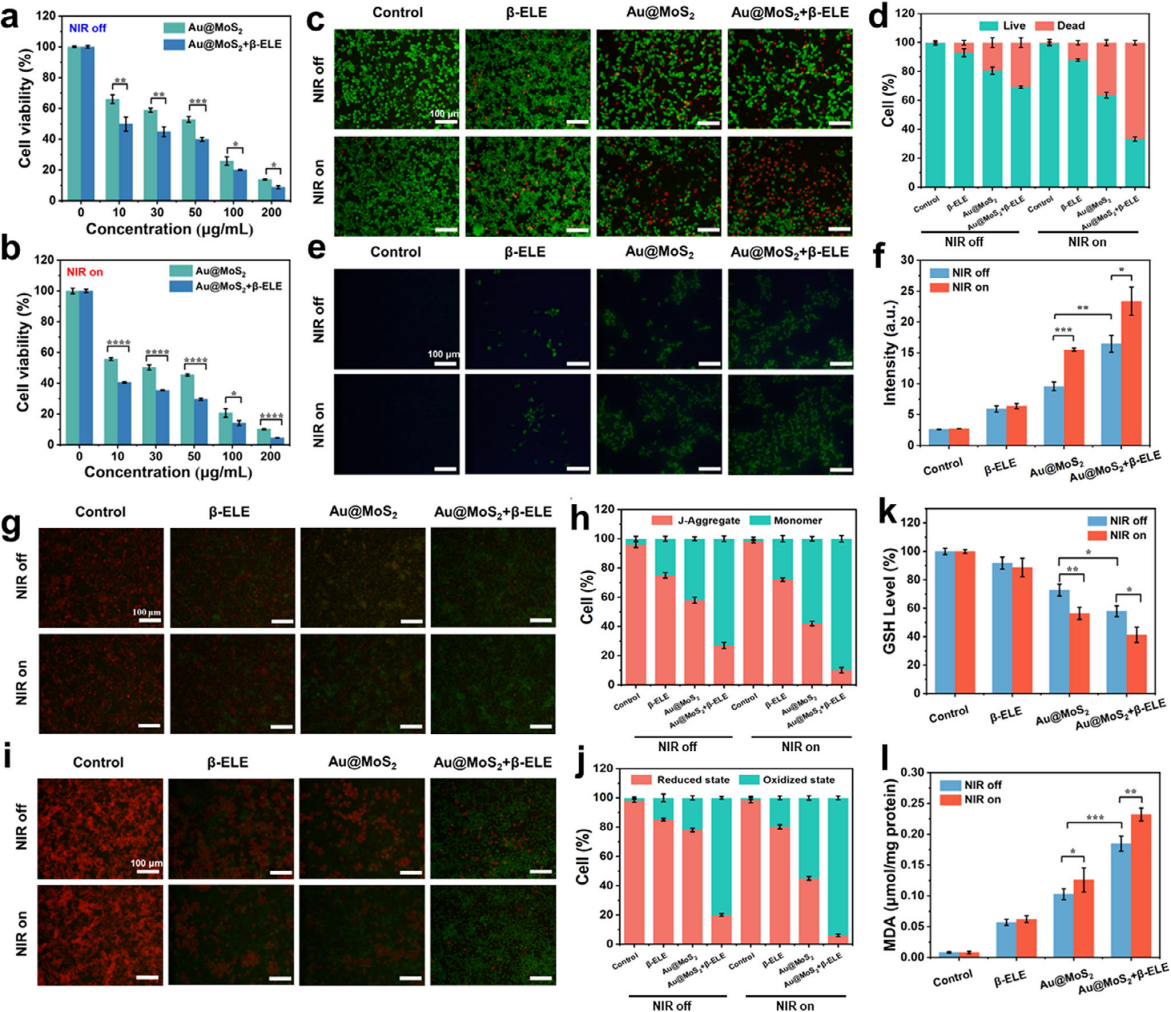
a) B16F10 cell viability after treatment with different concentrations of Au@MoS_2_ and Au@MoS_2_ + β‐ELE. b) B16F10 cell viability after treatment with different concentrations of Au@MoS_2_ and Au@MoS_2_ + β‐ELE under NIR laser irradiation (808 nm, 1.0 W/cm^2^). c) Fluorescence microscopy images of B16F10 cells after different treatments using Calcein‐AM/PI staining probes. d)  Quantitative analysis of live/dead staining percent in different groups.  e) Fluorescence microscopy images of B16F10 cells after different treatments showing ROS level using DCFH‐DA as the fluorescent probe. f) Mean fluorescence intensity of intracellular ROS level after different treatments. g) Fluorescence microscopy images of B16F10 cells after different treatments and staining with JC‐1. h) Quantitative analysis of J‐aggregate/monomer percent after different treatments. i) Fluorescence microscopy images of B16F10 cells after different treatments and staining with C11‐BODIPY581/591. j) Quantitative analysis of reduced/oxidized state percent after different treatments.  k) Relative GSH level in B16F10 cells after different treatment. l) MDA content in B16F10 cells after different treatment. Data are presented as mean ± S.D. (*n*  = 3).

The anti‐tumor effects of the combination of Au@MoS_2_ and β‐ELE are then evaluated at the cellular level. First, the anti‐melanoma effect of β‐ELE is evaluated by co‐incubating B16F10 cells with different concentrations of β‐ELE and the cell viability is determined by methylthiazolyldiphenyl‐tetrazolium bromide (MTT) assay. Among them, the NIR group is exposed to extra irradiation with 808 nm NIR laser (1 W cm^−2^) for 5 min. As shown in Figure S14 (Supporting Information), the killing rate of tumor cells is significantly enhanced with increasing β‐ELE concentration. Under NIR laser irradiation, the cell survival rate of β‐ELE group is not significantly different from that of the blank group, indicating that laser irradiation alone cannot enhance the therapeutic effect of β‐ELE. Furthermore, the therapeutic effect of BPNzyme is investigated after combination with β‐ELE. As indicated in **Figure** **4**a, Au@MoS_2_ and Au@MoS_2_ + β‐ELE show significant cytotoxicity and concentration‐dependent inhibition of B16F10 cell proliferation, in which the cell‐killing ability of Au@MoS_2_ + β‐ELE group is higher than that of the Au@MoS_2_ group at the same concentration. In addition, the viability of B16F10 cells is further reduced in the NIR laser irradiation group, indicating that the photothermal effect mediated by Au@MoS_2_ BPNzyme could synergistically inhibit the growth of tumor cells (Figure 4b). For comparison, as shown in Figure S15 (Supporting Information), MoS_2_ nanosheets exhibit dose‐dependent cytotoxicity, and their ability to kill tumor cells is significantly lower than that of the Au@MoS_2_ group at the same dose, confirming that enhanced catalytic activity and photothermal conversion capacity are key to improving antitumor efficacy.

Moreover, toxicity tests on normal skin cells (NIH3T3) show that the viability is still higher than 80% after treatment with a higher concentration of BPNzyme (500 µg mL^−1^), suggesting that BPNzyme can selectively kill tumor cells (Figure S16, Supporting Information). After combination therapy with Au@MoS_2_ and PTT, the same therapeutic effect can be achieved as the group using β‐ELE (100 µg mL^−1^) alone. Notably, the dose of β‐ELE in the combination therapy can be reduced by more than half, demonstrating the superiority of the combination therapy. Then, Calcein‐AM and PI staining is applied to label live cells (green) and dead cells (red) after different treatments. As shown in Figure 4c,d, the Au@MoS_2_ + β‐ELE group shows the brightest red fluorescence among four groups, suggesting that Au@MoS_2_ + β‐ELE has the strongest cytotoxic effect on B16F10 cells. In addition, under photothermal stimulation, the Au@MoS_2_ and Au@MoS_2_ + β‐ELE groups present more dead cells, which can be attributed to the good photothermal responsiveness of Au@MoS_2_ BPNzyme.

The main mechanism underlying the induction of ferroptosis in tumor cells is lipid peroxidation and apoptosis caused by oxidative stress.^[^ ^13^ ^]^ To validate the intracellular ROS generation mediated by Au@MoS_2_ + β‐ELE, ROS detection is performed using the fluorescent probe 2′,7′‐dichlorofluorescin diacetate (DCFH‐DA). As shown in Figure 4e,f, green fluorescence signal can be observed in the Au@MoS_2_, β‐ELE, and Au@MoS_2_ + β‐ELE groups, in which Au@MoS_2_ + β‐ELE group showing stronger ROS induction. Additionally, under NIR laser irradiation, the Au@MoS_2_ + β‐ELE group exhibits the strongest green fluorescence, indicating that photothermal stimulation can induce more ROS generation by Au@MoS_2_ + β‐ELE. On the contrary, MoS_2_ has a significantly weaker ability to induce ROS generation due to its lower POD‐like activity and photothermal performance (Figure S17, Supporting Information).Ferroptosis can lead to severe mitochondrial damage in tumor cells.^[^ ^29b^ ^]^ The mitochondrial membrane potential is then assessed using the JC‐1 probe. Under normal membrane potential conditions, the JC‐1 probe accumulates to be J‐aggregate and shows red fluorescence. However, when the mitochondria are damaged, the potential drops and the JC‐1 probe becomes monomeric with green fluorescence. As shown in Figure 4g,h, compared with β‐ELE, the green fluorescence intensity in the Au@MoS_2_ and Au@MoS_2_ + β‐ELE groups are enhanced under photothermal stimulation, indicating that Au@MoS_2_+ β‐ELE + NIR‐mediated combination therapy can further reduce mitochondrial membrane potential, thereby amplifying the anti‐tumor effect. LPO in cells is a critical step in the execution of ferroptosis.^[^ ^15a^ ^]^ The fluorescent probe C11 BODIPY 581/591 is used to detect LPO in B16F10 cells. The non‐oxidized C11 BODIPY 581/591 has red fluorescence, while the oxidized form possesses green fluorescence. As shown in Figure 4i,j, the red fluorescence of the probe gradually shifts to green fluorescence from the control, β‐ELE, Au@MoS_2_ to the Au@MoS_2_ + β‐ELE groups, indicating that Au@MoS_2_ and β‐ELE can trigger ferroptosis in tumor cells by inducing LPO production. Besides, under NIR laser irradiation, the Au@MoS_2_ + β‐ELE group exhibits stronger green fluorescence, suggesting that photothermal stimulation can promote lipid peroxidation in B16F10 cells, further promoting ferroptosis.

BPNzyme can effectively promote LPO accumulation through POD‐like catalytic reactions. However, high levels of GSH in the TME generally block this process, so we further assess the intracellular GSH levels after different treatments. As shown in Figure 4k, the levels of GSH in B16F10 cells from the Au@MoS_2_ + β‐ELE group are lower than those in the Au@MoS_2_ and β‐ELE groups. After application of 808 nm laser irradiation, the GSH depletion performance of the Au@MoS_2_@β‐ELE group can be further improved. The above results suggest that BPNzyme has synergistic NIR photothermal‐enhanced in vitro GSHOx‐like catalytic performance, which can effectively accelerate the depletion of intracellular GSH and amplify the effect of ferroptosis. Similar results can be found in the malondialdehyde (MDA) assay experiments. MDA is a natural product of lipid oxidation in living organisms, which occurs when oxidative stress occurs in animal or plant cells, and the level of LPO can be quantified by detecting the level of MDA.^[^ ^18^ ^]^ As shown in Figure 4l, the Au@MoS_2_ + β‐ELE + NIR group shows the highest level of MDA in the tumor cells, further confirming that the combination therapy can induce high accumulation of LPO in B16F10 cells. All the above results indicate that the combination therapy developed based on Au@MoS_2_ and β‐ELE has excellent nanocatalytic/photothermal/TCM synergistic anti‐tumor effects.

To further confirm the cell death pathways, B16F10 cells are pretreated with the ferroptosis inhibitor ferrostatin‐1 (FER‐1, 10 µM) for 1 h prior to treatment with either Au@MoS_2_ or Au@MoS_2_ + β‐ELE, followed by MTT assays. As shown in Figure S18 (Supporting Information), the results demonstrate that tumor cell death in both treatment groups occurs primarily through ferroptosis, as FER‐1 pretreatment effectively reduced the cytotoxicity of both BPNzyme and BPNzyme + β‐ELE, reversing the therapeutic trend. However, the anticancer effects were not completely abolished by FER‐1, indicating the involvement of additional cell death mechanisms beyond ferroptosis. To investigate the potential contributions of apoptosis and necrosis, we performed staining using the YO‐PRO‐1/PI apoptosis and necrosis detection kit.^[^ ^55^ ^]^ YO‐PRO‐1 is a green fluorescent DNA dye that selectively enters apoptotic cells due to their compromised membranes, while PI stains necrotic cells by binding to nucleic acids and emitting red fluorescence upon membrane rupture. As shown in Figure S19 (Supporting Information), control and control + NIR groups show no red or green fluorescence, indicating viable cells. β‐ELE treatment induces both green (apoptotic) and red (necrotic) fluorescence. Cells treated with Au@MoS_2_ + NIR exhibit predominantly red fluorescence with minimal green signal, suggesting necrosis as the primary death mechanism. In contrast, the Au@MoS_2_+ELE combination group displays overlapping red and green fluorescence (appearing orange‐yellow), demonstrating concurrent induction of both necrosis and apoptosis. These results confirm that the combined therapy under NIR irradiation maximizes tumor cell killing by simultaneously triggering multiple death pathways.

### 2.5 Mechanism Analysis of B16F10 Cells After Synergistic Therapy

**Figure 5:**
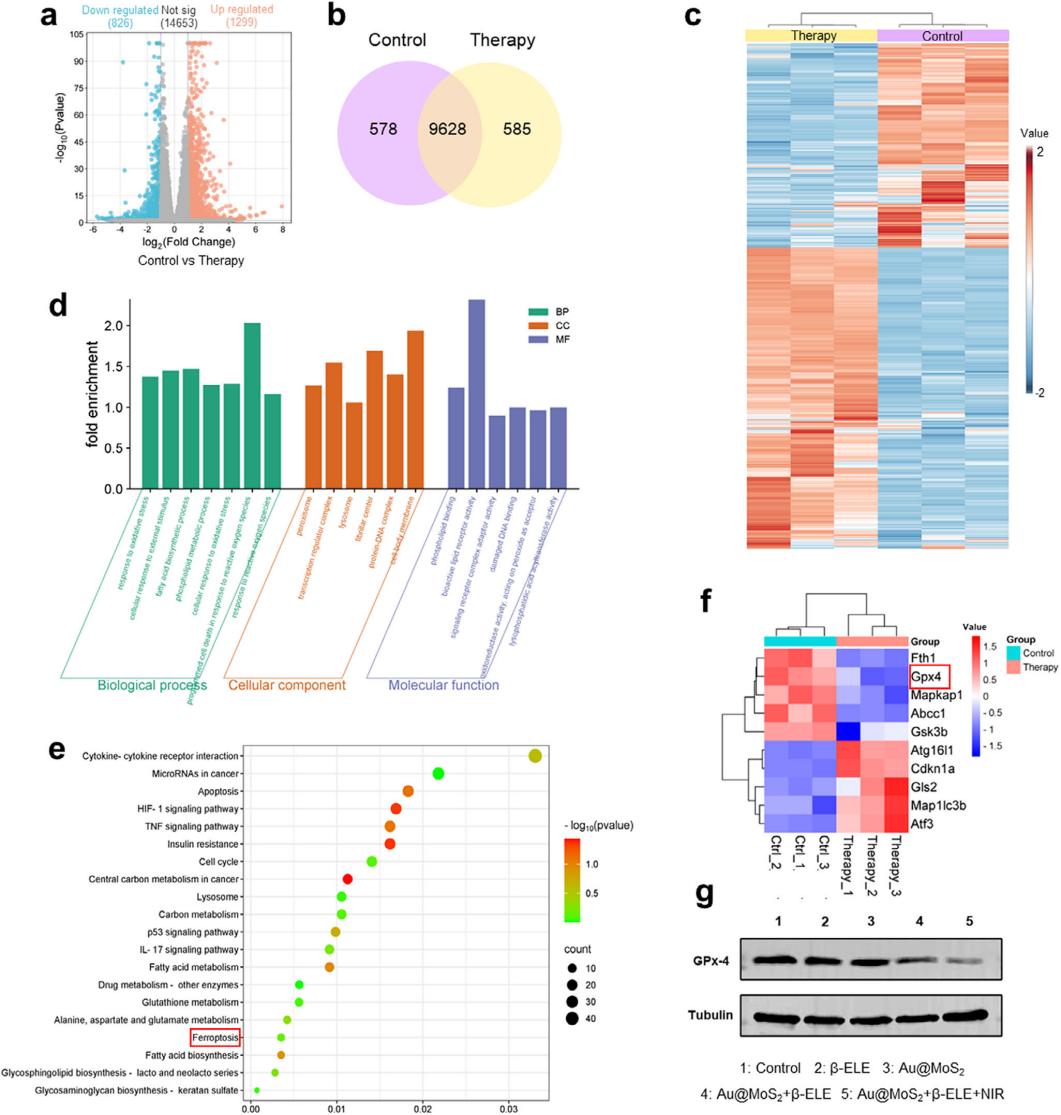
a) Volcano plot depicting the significant differences between the Au@MoS_2_ + β‐ELE + NIR group and the control group. b) Venn diagram showing primary differentially expressed genes of control and therapy groups. c) Heatmap of differentially expressed genes between the the Au@MoS_2_ + β‐ELE + NIR group and the control group. d) GO enrichment analysis of the gene functions analysis in the Au@MoS_2_ + β‐ELE + NIR versus control group. e) KEGG pathway enrichment analysis of the gene functions (Top 20). f) Heat map of major differentially expressed genes associated with ferroptosis pathway. g) Western blotting analysis of the GPx‐4 protein expression after different treatments.

The above evaluation of ferroptosis‐related indicators confirms that the combination therapy can achieve an efficient antitumor effect through the activation of the ferroptosis pathway. To further explore the underlying antitumor molecular mechanisms, transcriptomic analysis is used to reveal the differential gene expression profile of Au@MoS_2_ + β‐ELE + NIR‐treated B16F10 cells. First, the volcano plot analysis is conducted on the differentially expressed genes (DEGs). As shown in **Figure** **5**a, compared to the control group, 1299 genes are upregulated, and 826 genes are downregulated in B16F10 cells after combined therapy. As shown in Figure 5b, Venn diagram analysis further demonstrates the differences in gene expression between the control and treatment groups. Moreover, unsupervised hierarchical clustering analysis reveals that genes from the control and treatment groups are well‐clustered and distinct, indicating significant differences between two groups and confirming the high reliability of the transcriptomic data (Figure 5c). Subsequently, Gene Ontology (GO) and Kyoto Encyclopedia of Genes and Genomes (KEGG) pathway enrichment analyses are performed based on the RNA sequencing data. As shown in Figure 5d, GO analysis reveal that the combined treatment significantly alters biological processes, cellular components, and molecular functions in B16F10 cells. Notably, several significantly altered pathways are mainly associated with ROS and lipid metabolism, indirectly confirming key signal pathways associated with ferroptosis.^[^ ^14a^ ^]^ Additionally, KEGG enrichment analysis indicates that the Au@MoS_2_ + β‐ELE + NIR treatment group primarily induces B16F10 cell death through the activation of several ferroptosis‐related signaling pathways, such as HIF‐1, TNF, apoptosis, and glutamate metabolism (Figure 5e). Specifically, HIF‐1 regulates iron storage and uptake by modulating the expression of ferritin and transferrin receptor genes, thereby influencing ferroptosis.^[^ ^56^ ^]^ TNF promotes ferroptosis by activating NF‐κB and JNK signaling pathways, leading to disrupted iron metabolism and elevated ROS levels.^[^ ^57^ ^]^ ROS generated during ferroptosis can activate apoptotic signaling pathways, while mitochondrial dysfunction during apoptosis may conversely promote ferroptosis.^[^ ^58^ ^]^ Glutamate participates in glutathione synthesis, which clears ROS to inhibit ferroptosis. Disrupted glutamate metabolism reduces glutathione levels and increases cellular susceptibility to ferroptosis.^[^ ^59^ ^]^ The enrichment of these closely related pathways strongly supports the occurrence of ferroptosis.

The analysis of ferroptosis‐related gene expression shows that several differentially expressed genes, such as Map1lc3b, Slc7a11 and Atf3, are significantly upregulated, which effectively increases intracellular LPO and MDA levels, thereby inducing efficient ferroptosis (Figure 5f).^[^ ^60^ ^]^ In addition, the combination therapy effectively induced the conversion of GSH to GSSG and inhibited the expression of GPx‐4, thereby enhancing the ferroptosis effect in B16F10 cells. The above genomic analysis further demonstrates that the differentially expressed genes after treatment are closely related to the ferroptosis pathway, highlighting the critical role of combination therapy in modulating ferroptosis and treating melanoma. Furthermore, measurement of GPx‐4 protein expression in B16F10 cells after treatment shows a trend consistent with cell killing effects. The Au@MoS_2_@β‐ELE + NIR group shows the lowest GPx‐4 expression, resulting in improved ferroptosis of tumor cells (Figure 5g; Figure S20, Supporting Information).^[^ ^14a^ ^]^ These results provide valuable insights into the mechanism of melanoma therapy and demonstrate the significant potential of the multifunctional nanoplatform based on BPNzyme combined with TCM in activating ferroptosis for the treatment of melanoma.

### 2.6 Preparation and Characterization of Au@MoS_2_ + β‐ELE MN

Numerous studies have highlighted the potential of polymeric MN as a promising non‐invasive approach for the treatment of melanoma.^[^ ^8b^ ^]^ Recently, MN systems have been previously explored for the transdermal delivery of β‐ELE.^[^ ^61^ ^]^ As noted in prior studies, MN offers distinct advantages over conventional administration routes.^[^ ^62^ ^]^ First, unlike oral or intravenous delivery where drugs undergo systemic circulation and premature degradation before reaching target lesions, MN systems bypass first‐pass metabolism, enabling precise drug deposition into deep melanoma tissues, thereby enhancing therapeutic efficacy. Second, compared to other administration routes, MN‐based therapies minimize drug dispersion to healthy tissues, reducing off‐target effects and improving patient survival. Additionally, MN achieves superior efficacy at lower doses than subcutaneous injections by concentrating drugs directly at tumor sites. Furthermore, MN facilitates synergistic drug co‐delivery and therapy, optimizing treatment outcomes while mitigating resistance risks.

**Figure 6:**
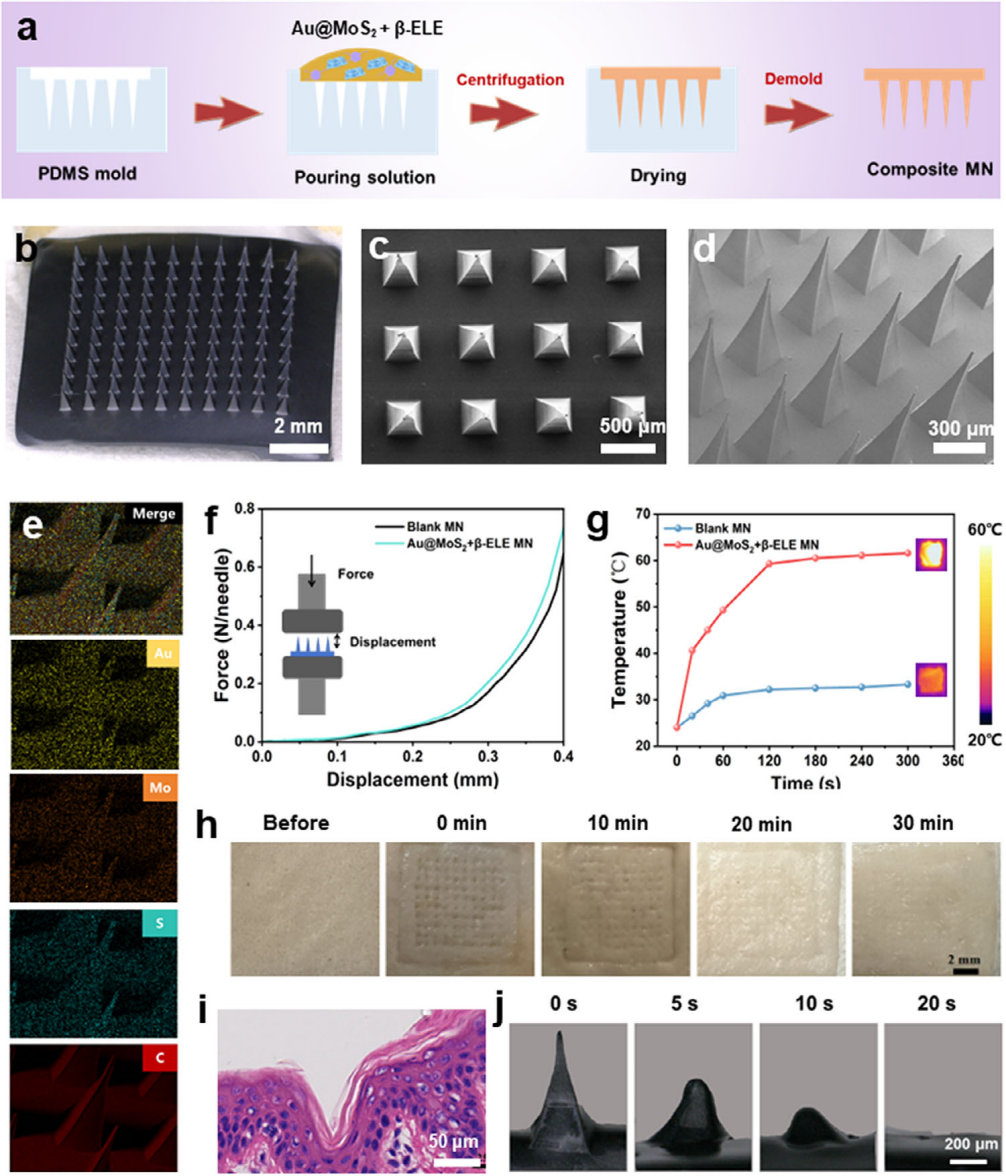
a) Schematic illustration of the manufacturing process for the Au@MoS_2_ + β‐ELE MN. b) Typical bright‐field microscopy image of Au@MoS_2_ + β‐ELE MN. Typical top‐view c) and cross‐section d) SEM images of Au@MoS_2_ + β‐ELE MN. e) Elemental mapping results of Au, Mo, S, and C elements in Au@MoS_2_ + β‐ELE MN. f) Mechanical strength test of blank MN and Au@MoS_2_ + β‐ELE MN. g) Photothermal property and relative thermal images of blank MN and Au@MoS_2_ + β‐ELE MN under NIR laser irradiation (808 nm, 1 W cm^−2^, 5 min). h) Photographs showing the recovery of pinhole within 30 min on porcine skin i) H&E staining of the rat skin after application of Au@MoS_2_ + β‐ELE MN. j) The bright‐field microscopy images of the dissolution process of Au@MoS_2_ + β‐ELE MN.

Inspired by the efficient ferroptosis induced by the combined Au@MoS_2_ + β‐ELE + NIR treatment group, we then use biocompatible and water‐soluble HA as MN matrix to further develop a MN patch integrated with Au@MoS_2_ BPNzyme and β‐ELE for in vivo treatment of melanoma. The toxicity of HA matrix materials on NIH3T3 cells is first analyzed by MTT assay (Figure S21, Supporting Information). The cytotoxicity results show that after co‐incubation with NIH3T3 cells using HA MN in the concentration range (0–500 µg mL^−1^) for 24 h, the survival rates are all above 85%, indicating that HA are slightly toxic to normal skin cells and exhibit good biocompatibility. As shown in **Figure** **6**a, the HA solution containing Au@MoS_2_ + β‐ELE is added to the polydimethylsiloxane (PDMS) mould and centrifuged to allow the hydrogel mixture to enter the needle tip, and then dried at 40 °C for 6 h. The composite MN is carefully disassembled from the mould after the MN was dried. The MN manufacturing method used in this study is cost‐effective and easy to perform.

The morphology of the fabricated HA MN and the Au@ MoS_2_ and β‐ELE intergated MN (Au@MoS_2_ + β‐ELE MN) is observed using a bright‐filed microscope. The tips of both MNs exhibit well‐ordered alignment, with an array size of 10 × 10 mm^2^ (Figure 6b; Figure S22, Supporting Information). As shown in Figure 6c,d, scanning electron microscope (SEM) images further reveal that the Au@MoS_2_ + β‐ELE MN exhibit aligned and well‐defined pyramidal shapes, with the tip height of 750 µm. Moreover, elemental mapping results of Au@MoS_2_ + β‐ELE MN show the uniform distribution of Au, Mo, S and C elements, further demonstrating the successful preparation of composite MN (Figure 6e).

After that, the mechanical strength of the prepared MN was tested. As indicated in Figure 6f, the average mechanical strength of Au@MoS_2_ + β‐ELE MN at a displacement of 400 µm is observed to be much greater than the minimum force (0.045 N) required to penetrate the stratum corneum, indicating that the mechanical strength of Au@MoS_2_ + β‐ELE MN is sufficient to penetrate the epidermal barrier.^[^ ^63^ ^]^ Meanwhile, the mechanical strength of the composite MN is slightly higher than that of the blank HA MN caused by the incorporation of Au@MoS_2_ BPNzyme. We further investigated the photothermal response of different MN. As shown in Figure 6g, the temperature of composite MN reaches up to 63 °C after 5 min of 808 nm laser irradiation, while the temperature of HA MN is only slightly higher than room temperature under the same condition, indicating that the heating rate of Au@MoS_2_ + β‐ELE MN is faster than that of HA MN, which is attributed to the good photothermal property of Au@MoS_2_ BPNzyme.

Next, the in vitro skin insertion ability of composite MN is visually assessed by puncturing porcine skin with Au@MoS_2_ + β‐ELE MN loaded with methylene blue dye (Figure S23, Supporting Information). As shown, microporous channels could be observed on the surface of porcine skin after insertion of the Au@MoS_2_ + β‐ELE MN, demonstrating that the composite MN can effectively penetrate epidermis. Then, the Au@MoS_2_ + β‐ELE MN is inserted in porcine skin, pressed for 5 min and then removed. The surface changes of the pig skin are observed at different time points, respectively. As shown in Figure 6h, the micropores on the porcine skin almost completely disappear after 30 min of composite MN application, indicating that MN can effectively form reversible microchannels on the skin. This reversible behavior highlights the potential of composite MN for efficient, non‐invasive skin penetration and drug delivery. Meanwhile, haematoxylin and eosin (H&E) staining assays reveal that Au@MoS_2_ MN can puncture the stratum corneum to form microchannels, further confirming that the composite MN could effectively penetrate the skin barrier to facilitate cargo delivery (Figure 6i). Furthermore, when the tip portion of the composite MN is placed in a PBS solution (pH 6.0), the tip of MN can completely dissolve within 20 s (Figure 6j), indicating that the composite MN has excellent dissolution properties. Besides, we successfully detected the signal peak from β‐ELE in the release solution using high‐performance liquid chromatography (HPLC), confirming the presence and effective release of β‐ELE in the composite MN (Figure S24, Supporting Information). Once inserted into the skin, the composite MN can dissolve rapidly to release Au@MoS_2_ BPNzyme and β‐ELE for efficient combination therapy of melanoma.

### 2.7 Evaluation of Anti‐Tumor Efficacy of Au@MoS_2_ + β‐ELE MN In Vivo

**Figure 7:**
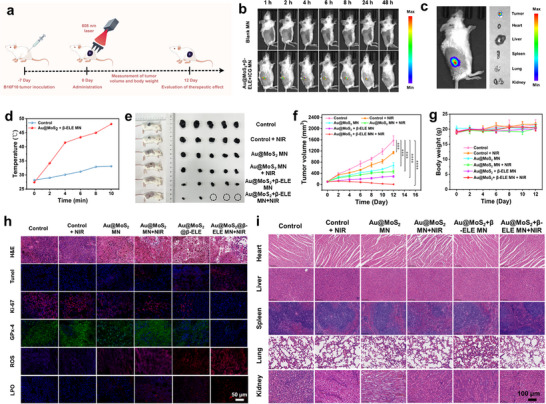
a) Schematic illustration of the timeline for in vivo antitumor experiment. b) In vivo fluorescence images of B16F10 tumor‐bearing mice after treatment with control group (blank MN) and Au@MoS_2_ + β‐ELE‐ICG MN for different time. c) Fluorescence images of the dissected organs and tumor tissues that collected after the Au@MoS_2_ + β‐ELE‐ICG MN treatment. d) Time‐dependent temperature curves of B16F10 tumor‐bearing mice with control group and Au@MoS_2_ + β‐ELE MN under NIR laser irradiation (808 nm, 0.5 W cm^−2^, 5 min). e) Photographs of tumor‐bearing mice as well as tumor tissues after 12‐day treatment. f) Time‐dependent change curve for tumor volume of different groups. g) Body weight change curve of different groups. h) Histological analysis including H&E, TUNEL, Ki‐67, GPx‐4, ROS, and LPO staining of tumor sections in different groups. i) H&E analysis of major organs (heart, liver, spleen, lung andkidney) from B16F10 tumor‐bearing mice after various treatment. Data are presented as mean ± S.D. (*n*  = 5).

Following the successful construction of the novel MN patch, the in vivo antitumor efficacy of Au@MoS_2_ + β‐ELE MN is further evaluated. B16F10 cells are first inoculated into BALB/c mice to establish tumor‐bearing mice models. After the tumor volume of mice had grown to 100 mm^3^, the mice are randomly grouped and subjected to different treatments. During the treatment process, MN are administered twice (Day 1 and Day 5). The tumor volumes and body weights are recorded every two days and the mice are sacrificed on Day 12 (**Figure** **7**a). To track the distribution of BPNzyme in vivo after MN administration, indocyanine green (ICG) is incorporated into Au@MoS_2_ to serve as a fluorescent probe in the tumor‐bearing mice. As shown in Figure 7b, the fluorescence signal from the MN composite group gradually increases over time, reaching a maximum at 4 h post‐administration, after which the signal begins to decrease and disappears completely at 48 h. In contrast, no significant distribution of the BPNzyme occurs in other organs, indicating that the MN‐delivered composites can effectively target the tumor site. Moreover, the mice are sacrificed and dissected after 1 h of MN administration. As shown in Figure 7c, no fluorescent signal could be observed in major organs (heart, liver, spleen, lungs, kidneys) outside the tumor, confirming that BPNzyme accumulates specifically at the tumor site with minimal impact on other organs. To investigate the temperature variation upon NIR laser irradiation after Au@MoS_2_ + β‐ELE MN is inserted into the tumor site of the mice, B16F10 tumor‐bearing mice are randomly divided into two groups (HA MN and Au@MoS_2_ + β‐ELE MN groups). The tumor sites are then irradiated with a laser (808 nm, 0.5 W cm^−2^) for 10 min, and the temperature changes in each group are recorded using an infrared thermography camera. As shown in Figure 7d, the HA MN group exhibits a slight temperature increase at the tumor site after 10 min of 808 nm laser irradiation (up to 33.4 °C), while the temperature in the tumor site of the Au@MoS_2_ + β‐ELE MN group gradually increases with prolonged laser irradiation. After 10 min of laser treatment, the tumor temperature in the Au@MoS₂ + β‐ELE MN group reaches 48.3 °C, which is sufficient to induce effective thermal damage.^[^ ^18^ ^]^

After combined therapy, the tumor in each group of mice after different treatment can be illustrated in Figure 7e, in which the tumors in the Au@MoS_2_ + β‐ELE MN + NIR group are almost completely eliminated, and this result is consistent with the tumor volume change curve (Figure 7f). This result indicates that Au@MoS₂ + β‐ELE MN group demonstrates the best in vivo antitumor efficacy under the assistance of NIR light stimulation. As shown in Figure 7g, no obvious decrease in body weight is observed in any of the groups throughout the treatment observation period. Furthermore, the H&E staining results show that the tumor cells in the control group maintain their morphology, whereas the tumor cells in Au@MoS_2_ + β‐ELE MN + NIR group show significant pathological changes and widespread cell necrosis (Figure 7h). These results indicate that the combination of BPNzyme, β‐ELE and PTT has a remarkable antitumor effect. Additionally, TUNEL and Ki‐67 immunofluorescence staining reveal that the Au@MoS_2_ + β‐ELE MN + NIR group exhibits the highest tumor cell killing activity and proliferation inhibition among six groups. The evident downregulation of GPx‐4 levels in the Au@MoS_2_ + β‐ELE MN + NIR group demonstrate that the excellent antitumor effect can be attributed to the occurrence of ferroptosis. Moreover, the accumulation of ROS and LPO in the treatment tissue validate high oxidative stress in tumors under the activation of synergistic ferroptosis. All the above results indicate that Au@MoS_2_ + β‐ELE MN under NIR irradiation have excellent in vivo anti‐melanoma performance.

To evaluate the potential biotoxicity of the proposed combination therapy, blood samples are collected from mice in each group at the end of treatment observation period, and then routine blood tests are then performed. As shown in Figure S25, no significant differences in the relevant indicators are observed between the treatment groups and the HA MN group, indicating that Au@MoS_2_ + β‐ELE MN exhibits good biocompatibility and no noticeable toxicity to the liver or kidney. Furthermore, H&E‐stained sections of major organs (heart, liver, spleen, lung and kidney) reveal no apparent pathological changes after treatment, further demonstrating that Au@MoS_2_ + β‐ELE MN possesses effectiveness and biosafety in melanoma therapy (Figure 7i).

## 3 Conclusion

In this work, we successfully developed a unique soluble MN patch integrated with the rationally designed BPNzyme and the TCM molecule β‐ELE for the synergistic amplification of ferroptosis‐mediated melanoma combination therapy. By LPE of MoS_2_ powder and the in situ self‐reduction reaction, the novel Au@MoS_2_ BPNzyme is easily and simply fabricated through a two‐step process. Based on the LSPR enhancement strategy, the rationally designed BNPzyme, as the key component of therapeutic system, not only exhibits excellent photothermal conversion capability but also significantly enhances dual enzyme‐like catalytic activities (POD and GSHOx) involved in the regulation of TME homeostasis. This Au@MoS_2_ BPNzyme can further combine with β‐ELE to synergistically activate the ferroptosis pathway in tumor cells. With the advantages of minimally invasive, painless, efficient and patient‐friendly features, the novel MN patch integrated with BPNzyme and β‐ELE effectively inhibit the growth of melanoma through a trimodal strategy of nanocatalytic/photothermal/TCM therapy, while showing good biocompatibility. In conclusion, this study contributes to a profound understanding of the regulation of the catalytic activity of heterogeneous bimetallic nanozymes as well as their modulation mechanism of TME for boosting ferroptosis. Beyond β‐ELE, other antitumor agents or ferroptosis inducers could also serve as promising candidates for combination therapy with the BPNzyme‐based MN platform in future studies. We believe the developed BPNzyme will expand the biomedical application of functional nanozyme for efficient combination therapy of tumors.

## 4 Experimental Section

### Preparation of MoS_2_ and Au@MoS_2_

To prepare Au@MoS_2_ BPNzyme, few‐layer 2D MoS_2_ nanosheets were first synthesized using a LPE method based on a top‐down strategy as previously reported.^[^ ^30a^ ^]^ Specifically, 500 mg of MoS_2_ powder was dissolved in a 45% ethanol solution and subjected to ultrasonication for 4 h using an ultrasonic probe in an ice bath. After centrifugation at 6000 rpm, the supernatant was collected and stored. Subsequently, MoS_2_ nanosheets solution was mixed with 100 µL of PVP (M_w_ = 40000, 1 mg mL^−1^) in a 10:1 ratio in 10 mL of deionized water. Then, 200 µL of HAuCl_4_ (14.5 mM) solution was rapidly added under stirring, and the mixture was stirred at room temperature for 30 min to obtain the Au@MoS_2_ solution. After washing by centrifugation with deionized water 2–3 times, the Au@MoS_2_ BPNzyme was stored at 4 °C for further use.

### Characterization of MoS_2_ and Au@MoS_2_

The morphology and size of the nanomaterials were characterized using TEM (HT‐7700). The structure and elemental distribution of Au@MoS_2_ were analyzed by HAADF‐STEM and energy‐dispersive X‐ray elemental mapping (Tecnai F30). The particle size and zeta potential were measured at 25 °C using a Zetasizer Lab (Nano ZS90, Malvern). The absorption spectra and various absorbances were analyzed using a UV—vis–NIR spectrophotometer (T Lambda 750, PerkinElmer). The thickness of the nanosheets was determined using AFM (Park Systems, NX10, Suwon). The elemental composition and chemical bonding properties of Au@MoS_2_ were characterized using XPS (Escalab 250Xi, Thermo). Raman spectroscopy (LabRam HR, Jobin Yvon) was used to characterize the material structure based on characteristic vibrational peaks. UV–vis diffuse reflectance spectroscopy (UV–vis DRS) was analyzed on a PerkinElmer Lambda 35 device.

### Photothermal Activity Evaluation

To investigate the in vitro photothermal heating behavior of different materials, water, same concentration of MoS_2_, and Au@MoS_2_ (100 µg mL^−1^)were placed in a 48‐well plate and irradiated with an 808 nm NIR laser. Infrared thermal images were captured at 0, 1, 2, 3, 4, and 5 min using an infrared thermal imager, and the temperature changes at different time points were recorded. Next, Au@MoS_2_ with various concentrations (0, 25, 100, 300 µg mL^−1^) were prepared and placed in a 48‐well plate. The samples were irradiated with an 808 nm NIR laser at a power density of 1.5 W cm^−2^. Then, Au@MoS_2_ aqueous solution was exposed to NIR laser (808 nm) at various power densities. To study the photothermal stability of Au@MoS_2_, the Au@MoS_2_ aqueous solution was exposed to an 808 nm NIR laser at a power density of 1.5 W/cm^2^ for 5 min, followed by natural cooling to room temperature. This process was repeated five times, and the temperature changes at different time points were recorded.

To study the photothermal conversion efficiency, the aqueous solutions of MoS_2_ and Au@MoS_2_ were exposed to an 808 nm NIR laser at a power density of 1.5 W cm^−2^ for 5 min, followed by natural cooling to room temperature. The temperature changes at different time points were recorded, and the photothermal conversion efficiency of MoS_2_ and Au@MoS_2_ was calculated. The photothermal conversion efficiency (η) is defined as the ability of the material to convert light energy into heat energy and is calculated using the previous methods.^[^ ^21a^ ^]^

### POD‐Like Nanozyme Activity Evaluation

To test the POD‐like catalytic ability of the materials, MoS_2_ and Au@MoS_2_ (200 µg mL^−1^) were introduced into a mixture of H_2_O_2_ and TMB (2 mL) and reacted for 5 min under acidic conditions (pH 5.5) at normal physiological temperature (37 °C). The absorption spectra of the three materials in the range of 300–800 nm were then measured using a UV—vis–NIR spectrophotometer. Additionally, the absorption values at 650 nm were measured after the reaction started under the same conditions.

To study the peroxidase‐like catalytic mechanism, the steady‐state kinetics of the simulated POD was analyzed using the Michaelis‐Menten equation based on the previous work.^[^ ^18^ ^]^ Specifically, H_2_O_2_ was used as the substrate, and its concentration was varied (0.01, 0.025, 0.05, 0.1, 0.25, 0.5, and 1 mM). MoS_2_ and Au@MoS_2_ were introduced into the mixture of H_2_O_2_ and TMB and reacted for 5 min under acidic conditions (pH 5.5) at 37 °C. The absorption values at 650 nm were measured. The specific values of K_m_ and V_max_ were determined by Lineweaver‐Burk plotting.

To investigate the catalysis of H_2_O_2_ decomposition into hydroxyl radicals by nanozymes, MoS_2_ and Au@MoS_2_ were introduced into. DMPO was then added to the a mixture of H_2_O_2_, TMB, and nanozyme, and reacted for 5 min under acidic conditions (pH = 5.5) at 37 °C. Then, EPR (EMX‐10/12, Bruker) was used to capture and detect hydroxyl radicals. To study the influence of NIR light on the POD‐like activity, one group was irradiated with an 808 nm NIR laser during the reaction, while the other group was not. After the reaction, the absorption spectra of the materials in the range of 300–800 nm were measured. To investigate the nanozyme activity of Au@MoS_2_ under different pH, temperature, and NaCl concentration conditions, Au@MoS_2_ was introduced into a mixture of H_2_O_2_ and TMB and reacted for 5 min under different conditions, and the absorption values at 650 nm were measured.

### GSHOx‐Like Nanozyme Activity Evaluation

To test the GSHOx‐like catalytic ability of the materials, MoS_2_ and Au@MoS_2_ (200 µg/mL) were introduced into a mixture of GSH and DTNB and reacted for 5 min under acidic conditions (pH = 5.5) at 37 °C. The absorption spectra of the three materials in the range of 400–500 nm were then measured using a UV—vis–NIR spectrophotometer. The absorption values at 412 nm were measured every 1 min using a UV—vis–NIR spectrophotometer after the reaction started. Subsequently, the K_m_ and V_max_ ​values of the nanozymes were obtained using the same testing method as for POD‐like activity.

### Preparation and Characterization of Au@MoS_2_ + β‐ELE MN

A mixed solution of β‐ELE and Au@MoS_2_ in a 1:1 ratio was prepared and mixed with 5% HA. The mixture was loaded into a preformed PDMS mold for preparing MN. After centrifuged at 3500 rpm for 15 min, the mixture then placed in an oven at 40 °C. After 6 h, the MN was carefully removed and stored in a desiccator. The morphology of the MN was characterized using a stereo microscope (Nikon SMZ745T, Japan) and SEM (Gemini‐300, ZEISS, Germany) with EDS (OXFORD Xplore 30, Oxford, UK). A universal testing machine was used to measure the mechanical properties of the MN. To study the photothermal properties of the MN, an 808 nm NIR laser and an infrared thermal imager were used to record the temperature changes of the MN. To study the solubility of the MN, the tips of the MN were placed in PBS solution at pH 5.5, and the dissolution of the MN tips was observed and recorded using a stereo microscope. Analysis of β‐ELE release from MN was carried out by a high‐performance liquid chromatography (HPLC, CTO‐10AS, Shimadzu). To study the in vitro skin insertion ability of the MN, the MN was inserted into pig skin, and the surface changes of the pig skin were observed and recorded at 0, 10, 20, and 30 min. Subsequently, H&E staining was used to further assess the in vitro skin insertion ability of the MN.

### Animal Studies

All animal experiments were conducted in a standard barrier environment of the Laboratory Animal Center of Hangzhou Normal University (Hangzhou, China) and were supervised and approved by the Animal Ethics Committee of Hangzhou Normal University (20231114‐02). To establish a melanoma mouse model, B16F10 cells in the logarithmic growth phase were digested with trypsin, collected by centrifugation, and diluted into a cell suspension of 1 × 10^6^ cells mL^−1^ in culture medium, which was then kept on ice. After that, 100 µL of the cell suspension was subcutaneously injected into the dorsal right upper limb of the mouse to establish a tumor‐bearing mouse model. The growth of the tumor was monitored every 2 days after the model was established. To study the biodistribution, ICG and Au@MoS_2_ were mixed in an aqueous solution by oscillation for 24 h, and then Au@MoS_2_+β‐ELE+ICG MN was obtained using the same steps as described above. Mice with tumor volumes of ≈100 mm^3^ were selected, and Au@MoS_2_+β‐ELE + ICG MN and HA MN were inserted into the mouse melanoma. The distribution of the nano‐drug in the tumor‐bearing mice was observed using an animal imager. To explore the in vivo photothermal therapy capability of BPNzyme, the temperature increase of the mouse tumor site after irradiation with NIR laser was recorded using an infrared thermal imager. After administration, the mice in each group were irradiated with an 808 nm laser at a power density of 0.5 W cm^−2^ for 10 min. The temperature changes of each group of mice were recorded at 0, 2, 4, 6, 8, and 10 min using an infrared thermal imager. Subsequently, the effects of different treatments on melanoma growth were compared. First, B16F10 tumor‐bearing mice with tumor volumes of ≈100 mm^3^ were randomly divided into six groups, with eight mice in each group: HA MN group, HA MN + NIR group, Au@MoS_2_ MN group, Au@MoS_2_ MN + NIR group, Au@MoS_2_+β‐ELE MN group, and Au@MoS_2_+β‐ELE MN + NIR group. Starting from the first day of administration, the body weight and tumor volume of the tumor‐bearing mice were measured every 2 days. The tumor volume was calculated using the following equation:

(1) $$V=L\times W^{2}/2$$

where V is the tumor volume, L is the tumor length, and W is the tumor width. After 12 days of administration, all mice were sacrificed. Representative mouse the tumor site was photographed. After cervical dislocation, the tumor tissues from each group of mice were collected. The tumor tissues were immediately washed with PBS, fixed overnight in 4% paraformaldehyde, embedded in paraffin, sectioned, and mounted on slides for H&E staining. The sections were scanned using an automated slide scanning imager to observe tumor necrosis. Additionally, the tumor tissues were subjected to TUNEL, Ki‐67, GPX‐4, ROS, and LPO immunohistochemical analyses.

### Statistical Analysis

Statistical analysis was performed using Origin 2021. Data are presented as mean ± standard deviation (Mean ± SD). Statistical differences were analyzed using t‐tests. The significance threshold was set at *p* < 0.05. *, **, ***, and **** indicate *p* < 0.05, *p* < 0.01, *p* < 0.001, and *p* < 0.0001, respectively.

## Conflict of Interest

The authors declare no conflict of interest.

## Supporting information



Supporting Information

## Data Availability

The data that support the findings of this study are available from the corresponding author upon reasonable request.
