# Supplementary material for: Bimetallic Plasmonic Nanozyme‐Based Microneedle for Synergistic Ferroptosis Therapy of Melanoma
Source: Adv Sci (Weinh). 2025 May 19;12(30):e04203. doi: 10.1002/advs.202504203 (PMC12376522; doi:10.1002/advs.202504203)
Supplement: Supplementary file 1 — Supporting Information [file ADVS-12-e04203-s001.docx]

Supporting Information

Bimetallic Plasmonic Nanozyme-Based Microneedle for Synergistic Ferroptosis Therapy of Melanoma

Wei Duan ^*^, Keying Xu^,^, Yue Gao, Sheng Huang, Xueqian Xia, Xiang Liu, Shuangxue Pan, Chunpeng Jiao, Weijian Cheng, Yong Guo, Jingwen Zhao^*^, Jia-Wei Shen^*^

**Supplementary Experimental Details**

**Bandgap energy calculation**

Experimentally, the bandgap energy is calculated from the UV–vis DRS absorption edge. Due to the unclear presence of the excitonic peak, the determination of the band-gap energy becomes imprecise and in such situations, the Tauc relation helps to estimate the energy of the band-gap. [1] The Tauc relation is given as,(Ahν)^1/2^=A(hν−Eg) where α, ν,h, Eg and A correspond to the absorption coefficient, frequency of photon, Planck’s constant, band-gap and proportionality constant, respectively.

**FDTD simulation**

FDTD simulations were performed using the software FDTD Solutions 8.19 (Lumerical Solutions, Inc). In the model of this study, the diameter of the Au nanoparticles was set to 20 nm, while the thickness and size of the MoS₂ were set to 5 nm and 200 × 200 nm, respectively. A mesh size of 0.5 nm was employed for accurate calculations, and the total simulation time was 1000 fs. A plane wave source with a wavelength of 808 nm was incident along the Z-axis and polarized along the X-axis. The distribution of the electromagnetic field in the XY plane was analyzed.

**DFT calculation**

Density functional theory (DFT) calculations were performed using the Vienna Ab Initio Simulation Package (VASP). The projector-augmented wave (PAW) method was employed to describe the interaction between core and valence electrons. The generalized gradient approximation (GGA) in the form of the Perdew-Burke-Ernzerhof (PBE) functional was used to treat electron exchange and correlation effects. The DFT-D3 method was applied for long-range van der Waals interactions. To ensure accuracy, the energy cutoff for the plane wave basis was set to 400 eV, and a Monkhorst-Pack grid of size 3×3×1 was adopted, with the residual force on each atom being below 0.05 eV/Å.

**Cell culture**

Mouse melanoma cells (B16F10) were cultured in RPMI-1640 medium containing 10% fetal bovine serum (FBS) and 1% penicillin (100 units/mL)-streptomycin (100 μg/mL) (PS). Mouse embryonic fibroblast cells (NIH3T3) were cultured in Dulbecco's Modified Eagle Medium (DMEM, Gibco) containing 10% FBS and 1% PS.All cells were maintained in a cell incubator at 37 °C under 5% CO_2_.

**Cell viability**

For the cell viability assay, B16F10 cells were evenly seeded into a 96-well plate (5 × 10^4^ cells per well) and incubated for 12 h. Different concentrations of materials and drugs were added and incubated for 24 h. Cell viability was assessed using a MTT assay. For FER-1 groups. B16 F10 cells were pretreated with FER-1 (10 μM in DMSO) for 60 min prior to different treatment according to previous work. [2]

**Live/Dead staining**

To visually depict the in vitro anti-tumor effects, cells were subjected to staining with Calcein-AM/Propidium Iodide (PI) and observed under an inverted fluorescence microscope. The  quantitative analysis of live/dead staining percent was conducted according to previous work.[3]

**Intracellular ROS evaluation**

For ROS detection, similar to the scheme for the imaging of living and dead cells, after B16F10 cells were submitted to different treatments, DCFH-DA was added and incubated for 30min. After the residual DCFH-DA is completely removed with PBS, the fluorescent stain images were obtained to estimate the level of intracellular ROS. The  quantitative analysis of ROS staining percent was conducted according to previous work. [3]

**Mitochondrial damage evaluation**

The fluorescent dye JC-1 was utilized to assess mitochondrial damage. Initially, B16F10 cells were treated with various groups, gently washed with PBS and then incubated with JC-1 for 30 min. Finally, the extent of mitochondrial damage was observed using an inverted fluorescence microscope.

**Intracellular GSH evaluation**

The assessment of intracellular GSH levels was conducted using a reduced GSH content assay kit. After different treatment, the cells were harvested, and the intracellular GSH levels were quantified following a standardized protocol.

**Intracellular MDA evaluation**

For MDA content assays, B16F10 cells were subjected to detection using a MDA measurement kit under various treatments.

**LPO evaluation**

For LPO evaluation, B16F10 cells were first seeded in 24-well plates (5ⅹ10^4^ cells per well, 1 mL of DMEM medium), followed by treating with different samples at 37 °C. Subsequently, cells were incubated with C11-BODIPY581/591 fluorescent probe (10 μM) for 30 min. The variation of LPO was shown as the red/green fluorescent signal under inverted fluorescence microscope system observation.

**Apoptosis and Necrosis Detection**

For evaluation of the apoptosis and necrosis, B16F10 cells were detected using an Apoptosis and Necrosis Detection Kit with YO-PRO-1/PI dye (Beyotime Biotechnology, China). The B16F10 cells cells with the different treatments were seeded in six-well plates and cultured for 24 h. The cells were then washed once with PBS, following which by 1 mL of staining solution was added and the cells were incubated at 37 °C in the dark for 20 min. Following this, the staining was observed under a fluorescence microscope.

**Western blot assay**

After different treatment, B16F10 cells were then homogenized in RIPA cell lysis buffer, and protein concentration was measured by a BCA kit. Equal amounts of protein were loaded into SDS-PAGE loading buffer, separated by electrophoresis, and transferred to PVDF membranes. The membranes were blocked with skim milk and incubated overnight at 4 ℃ with anti-GPx-4 (1: 8000). After washing with TBST solution, the membranes were incubated with secondary antibodies against rabbit IgG (1: 12000) or mouse IgG (1: 12000). Expression levels were normalized for minor differences in loading.

**Transcriptome sequencing analysis**

Initially, total RNA was extracted from cells using TRIzol reagent, and the RNA concentration and purity were determined using NanoDrop, ensuring that the A260/A280 ratio was between 1.8 and 2.1. The mRNA library was constructed using the Illumina TruSeq RNA Sample Preparation Kit. rRNA was first removed from the samples, followed by the capture of mRNA using magnetic beads and reverse transcription to synthesize cDNA, after which the cDNA library was amplified. The quality and fragment size of the library were assessed using the Agilent 2100 Bioanalyzer to ensure its suitability for subsequent sequencing. High-throughput sequencing was performed using the Illumina HiSeq platform. The cleaned sequences were aligned with the reference genome using the HISAT2 alignment tool to obtain the expression levels of each gene. Differential gene expression analysis was conducted using DESeq2 to screen for significantly differentially expressed genes with |log2 fold change| > 2 and p-value < 0.05. Gene ontology (GO), Kyoto Encyclopedia of Genes and Genomes (KEGG), and gene set enrichment analysis (GSEA) were performed on the differentially expressed genes, and these steps were completed by Novogene Company.

**Supplementary Figures and Tables**


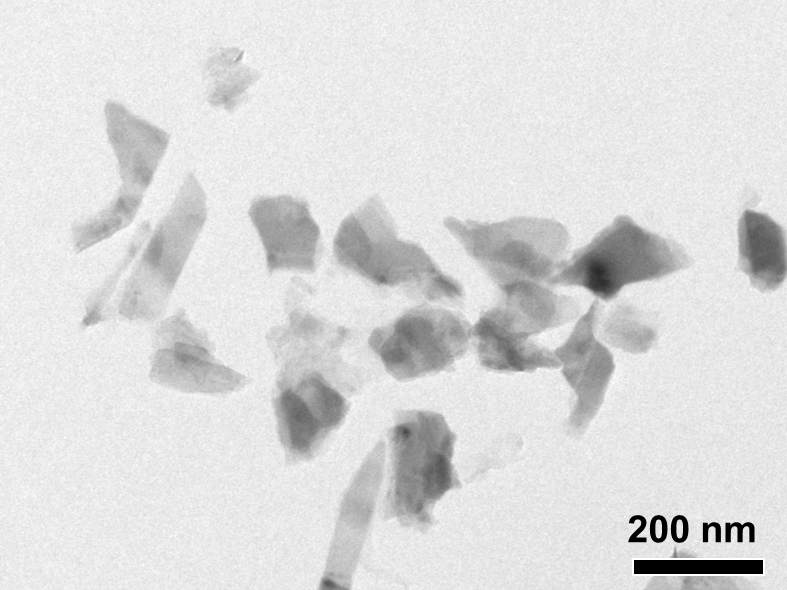


**Figure S1.** TEM image of MoS_2_ nanosheet.


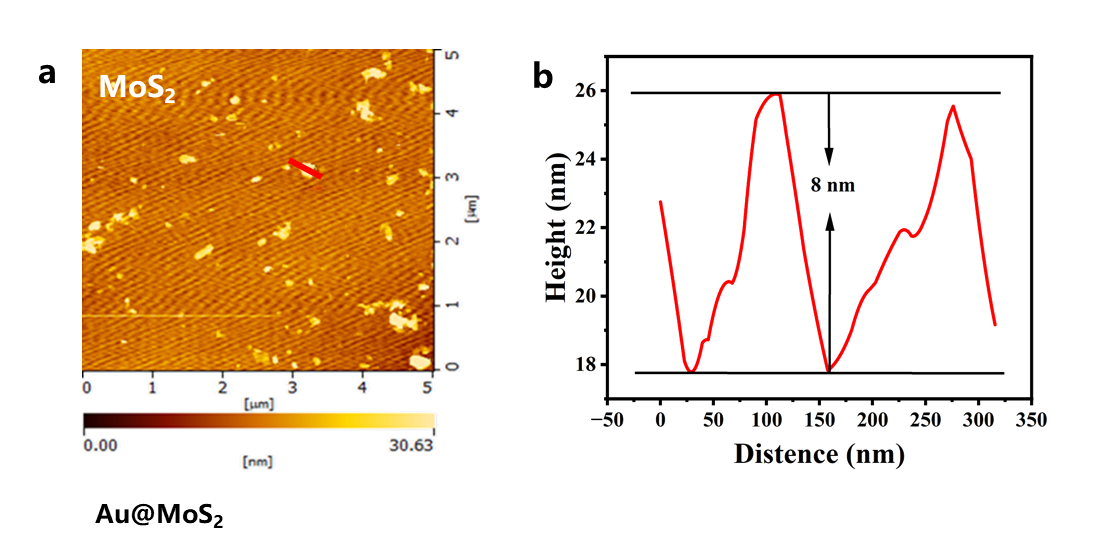


**Figure S2.** AFM image (a) and height measurement (b) of MoS_2_.


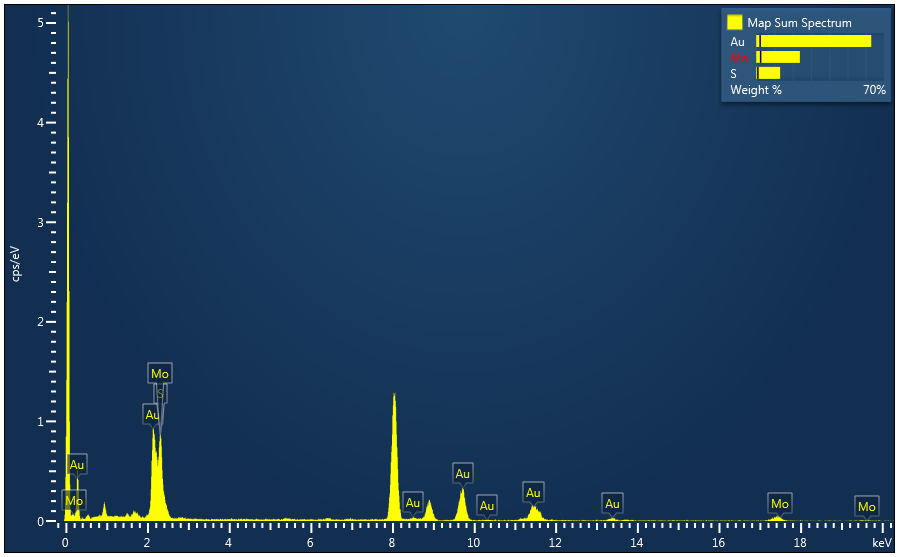


**Figure S3.** EDS results of Au@MoS_2_.


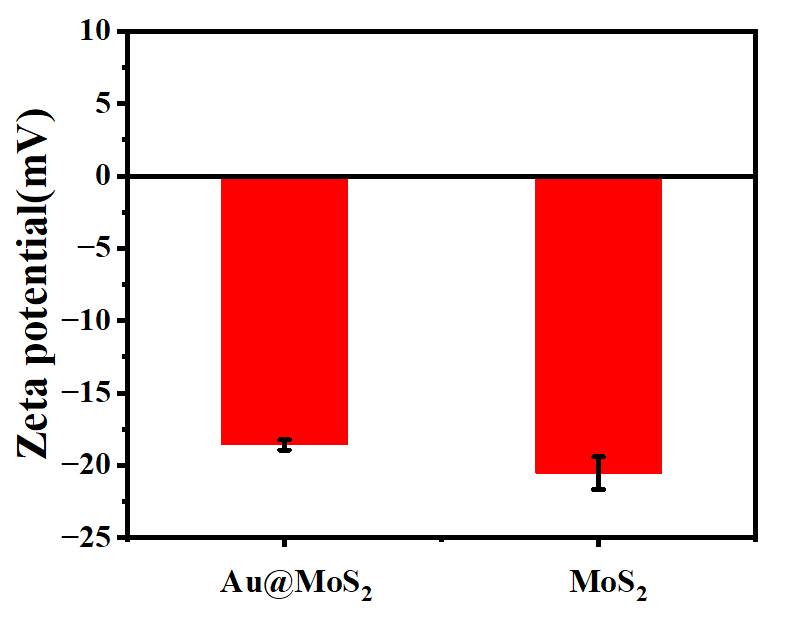


**Figure S4.** Zeta potential of MoS_2_ and Au@MoS_2_.


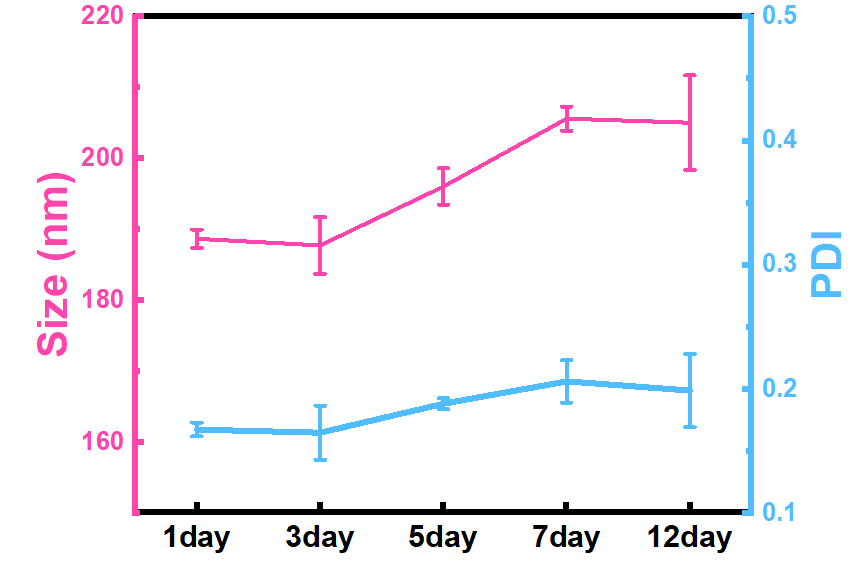


**Figure S5.** Size and PDI of Au@MoS_2_ over 12 days deterimend by DLS.


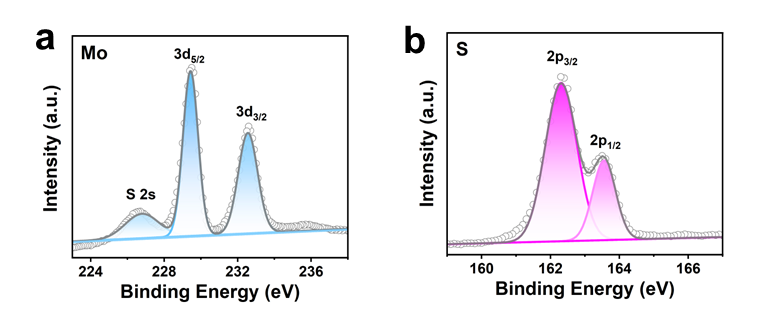


**Figure S6.** . High-resolution Mo 3d (a) and S 2p (b) XPS spectra of Au@MoS_2_.


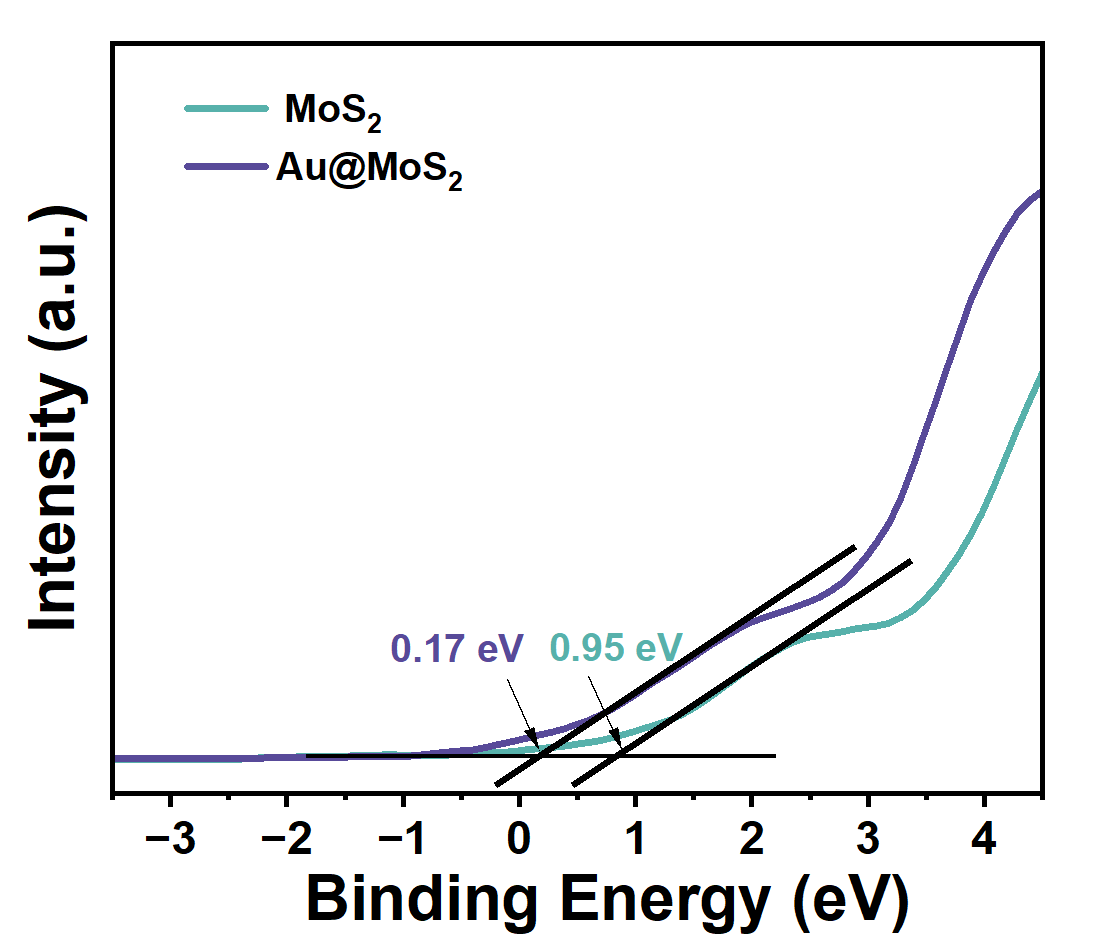


**Figure S7.** The XPS [valence band](https://www.sciencedirect.com/topics/chemistry/valence-band) spectra of MoS_2_ an Au@MoS_2_.


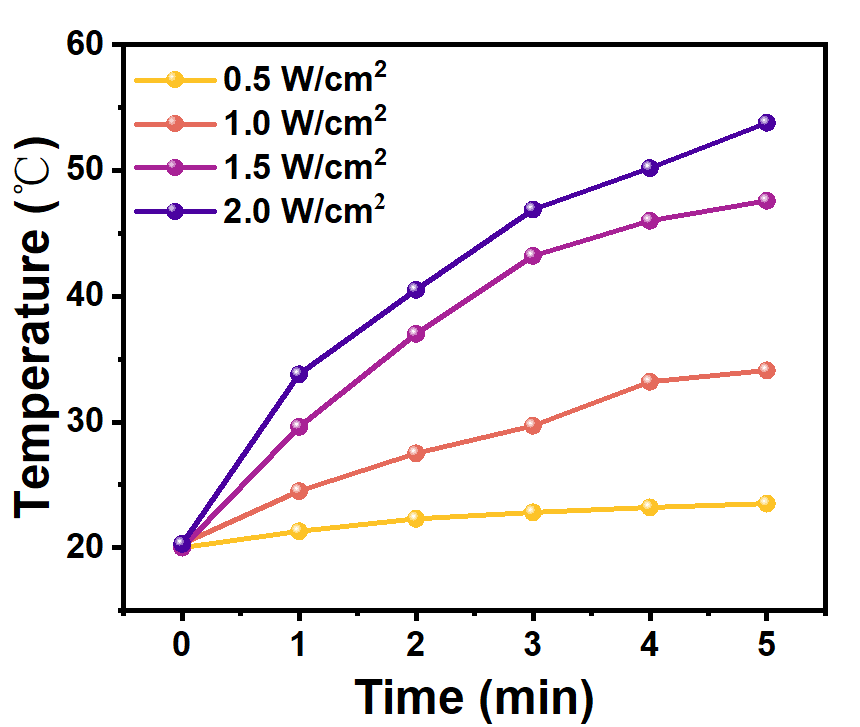


**Figure S8.** Temperature change curve of Au@MoS_2_ with different power intensity of NIR laser.


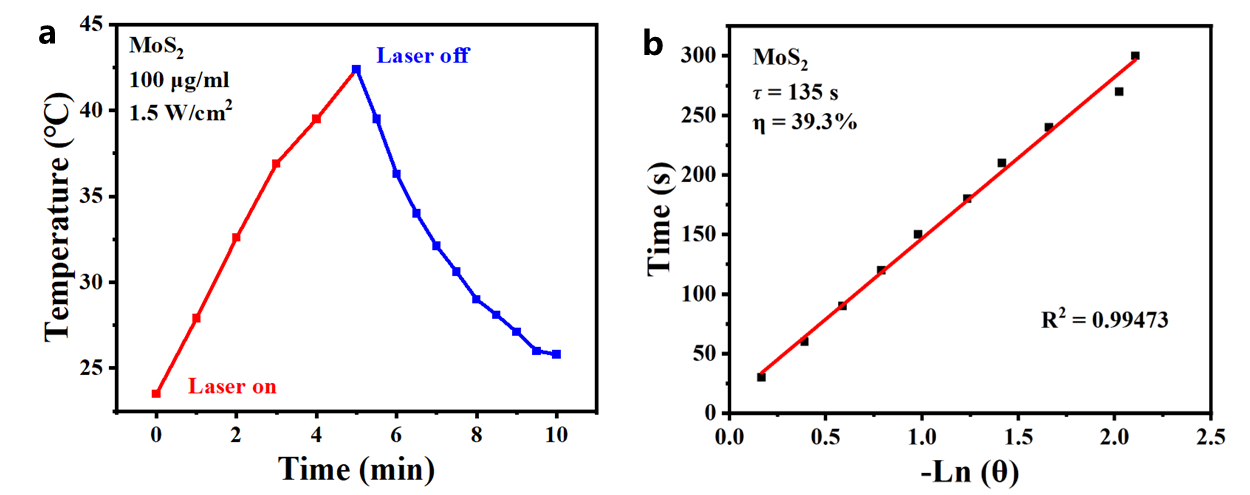


**Figure S9.** (a) Temperature change curve of MoS_2_ (100 μg/mL) with NIR irradiation (1.5 W/cm^2^) on and off. (b) The photothermal conversion efficiency obtained from the relationship of linear time data vs −Ln θ.


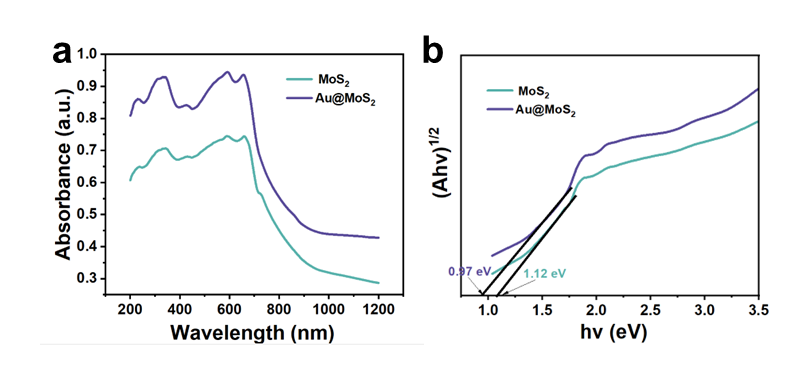


**Figure S10.** (a) The UV-DRS absorption spectrum of MoS_2_ and Au@MoS_2_. (b)  The plot of (Ahν)^1/2^ vs. energy hν and [bandgap energy](https://www.sciencedirect.com/topics/engineering/band-gap-energy) of MoS_2_ and Au@MoS_2_.


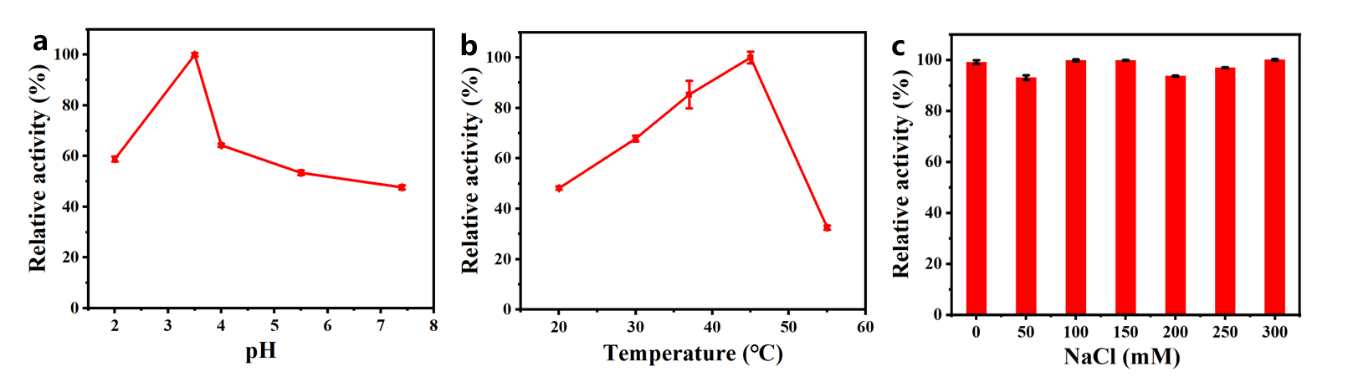
**Figure S11.** Effect of pH (a), temperature (b) and salt concentration (c) on the POD-mimic catalytic performance.


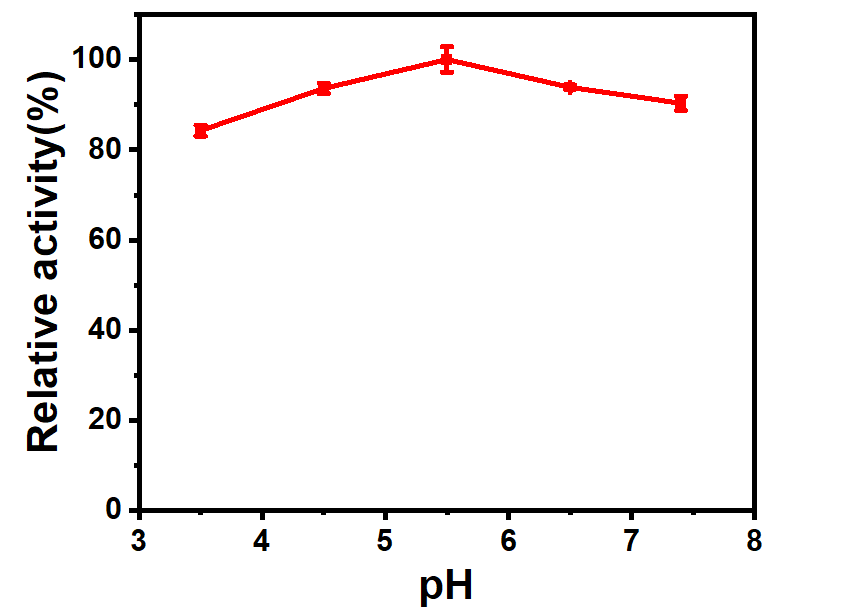


**Figure S12.** Effect of pH on the GSHOx-mimic catalytic performance.


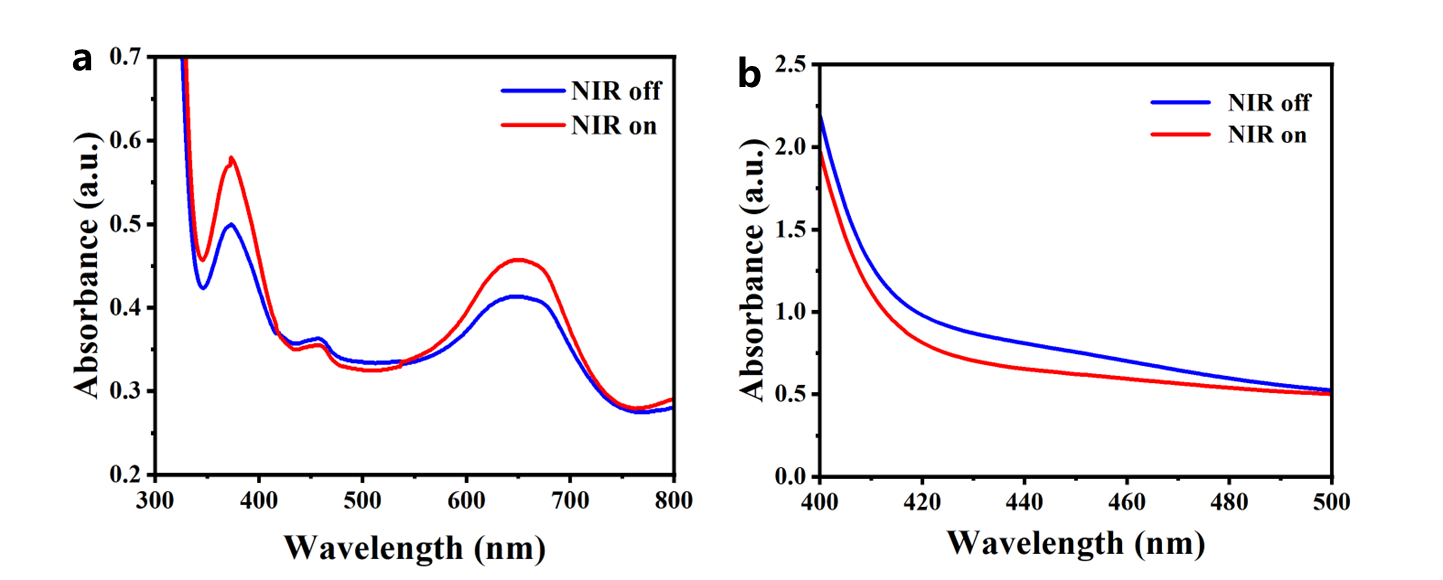


**Figure S13.** Influence of NIR laser irradiation on POD-like (a) and GSHOx-like (b) catalytic system.


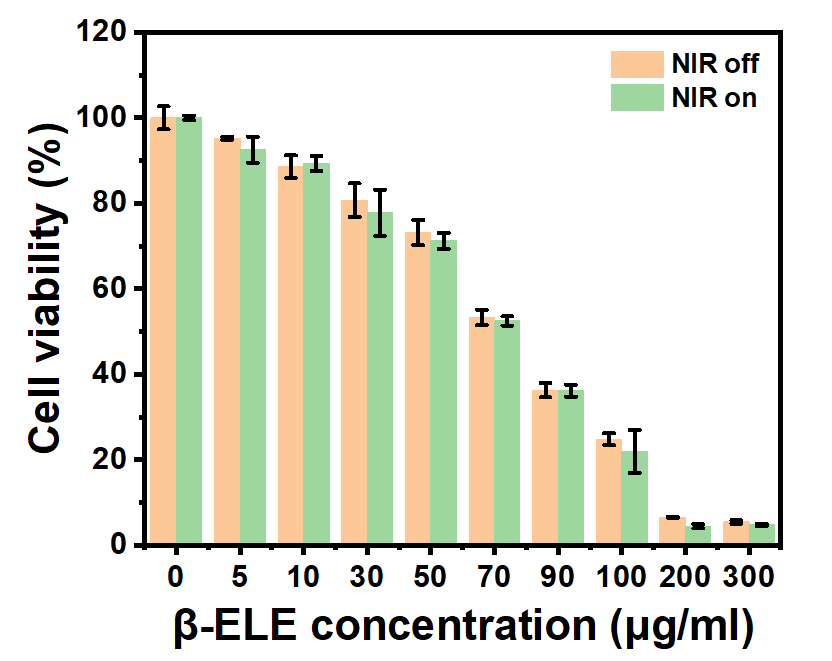


**Figure S14.** B16F10 cell viability after treatment with different concentrations of β-ELE with NIR laser irradiation off and on (808 nm, 1.0 W/cm^2^).


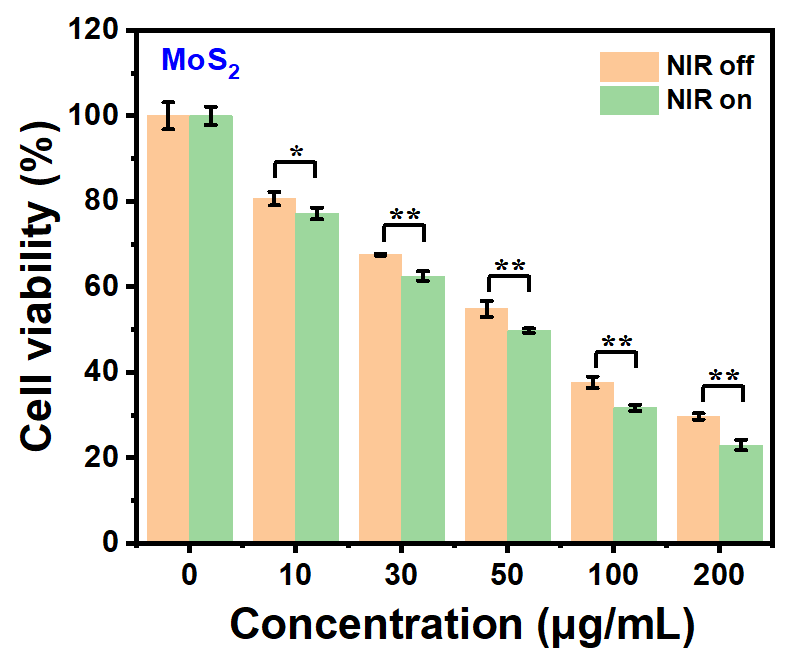


**Figure S15.** B16F10 cell viability after treatment with different concentrations of MoS_2_ with NIR laser irradiation off and on (808 nm, 1.0 W/cm^2^).


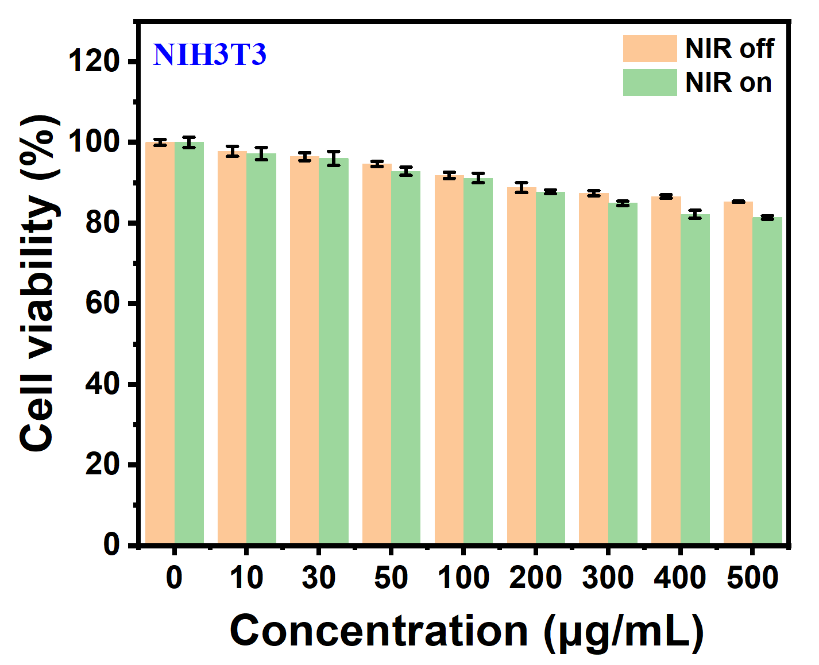


**Figure S16.** NIH3T3 cell viabilities after treated by different concentration of Au@MoS_2_.


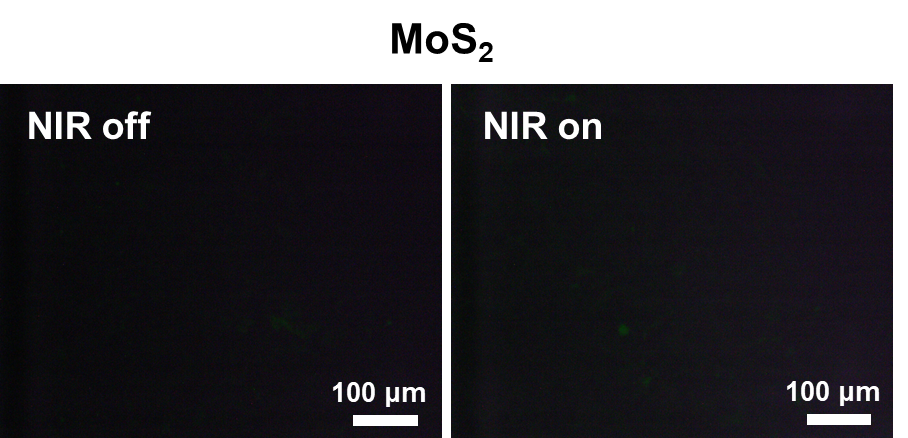


**Figure S17.** DCFH-DA staing fluorescent images of B16F10 cells after MoS_2_ treatment with NIR laser irradiation off and on (808 nm, 1.0 W/cm^2^).


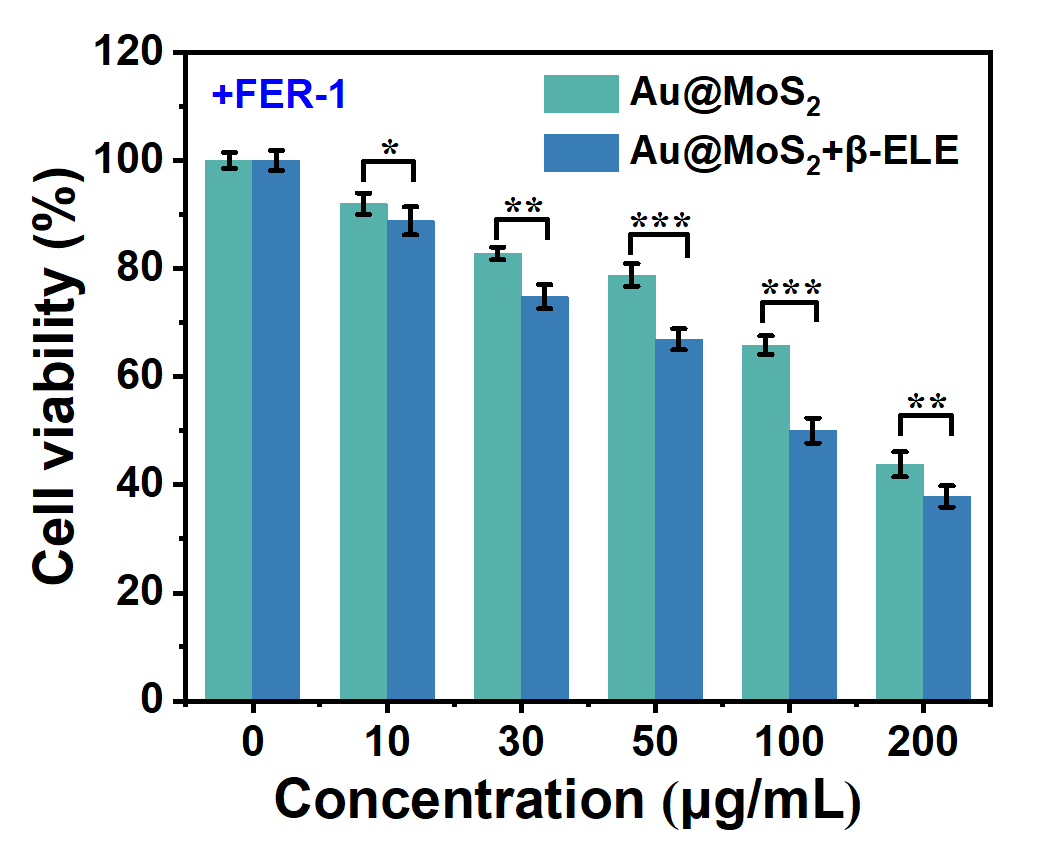


**Figure S18.** B16F10 cell viability pretreated with ferroptosis inhibitors (FER-1, 10 μM) for 1 h followed by Au@MoS_2_ and Au@MoS2+β-ELE treatment,.


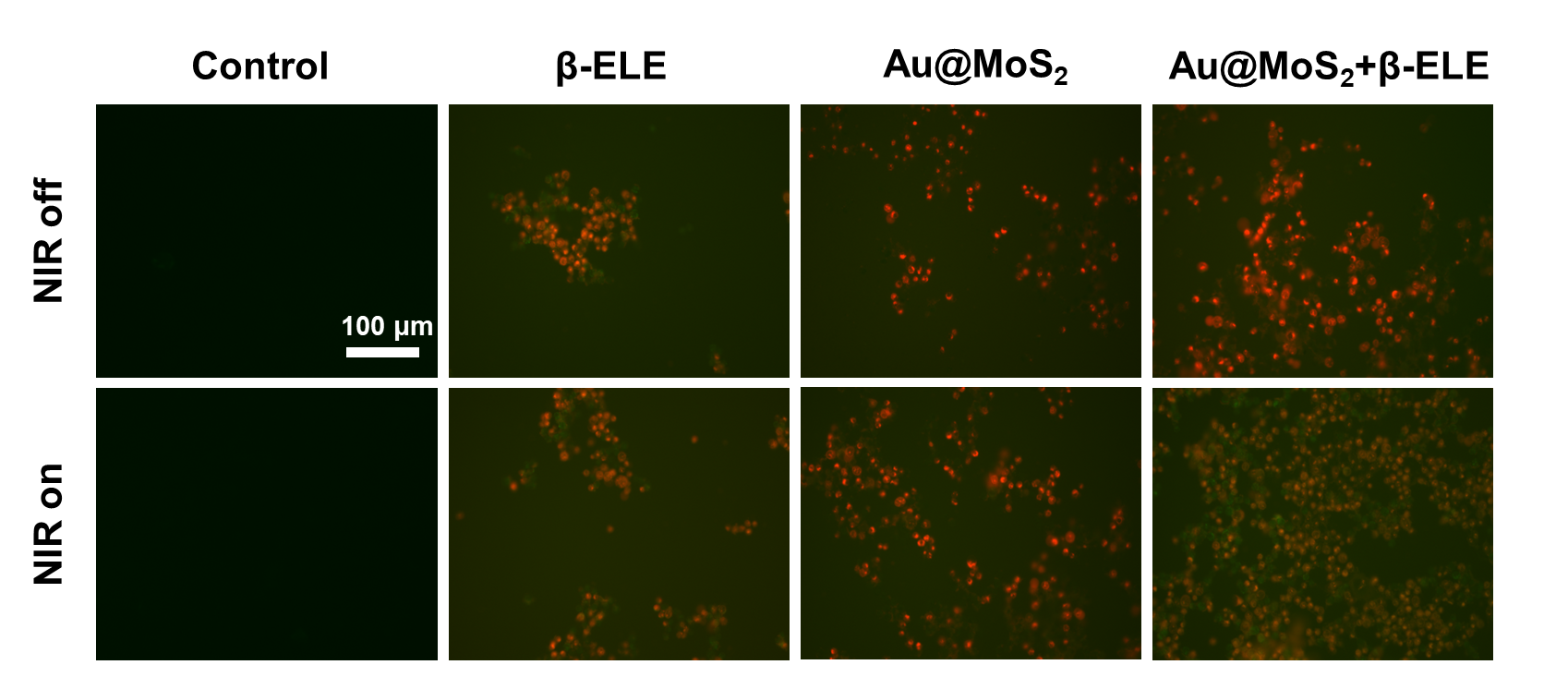


**Figure S19.**  YO-PRO-1/PI dye staing fluorescent images of B16F10 cells after different treatments.


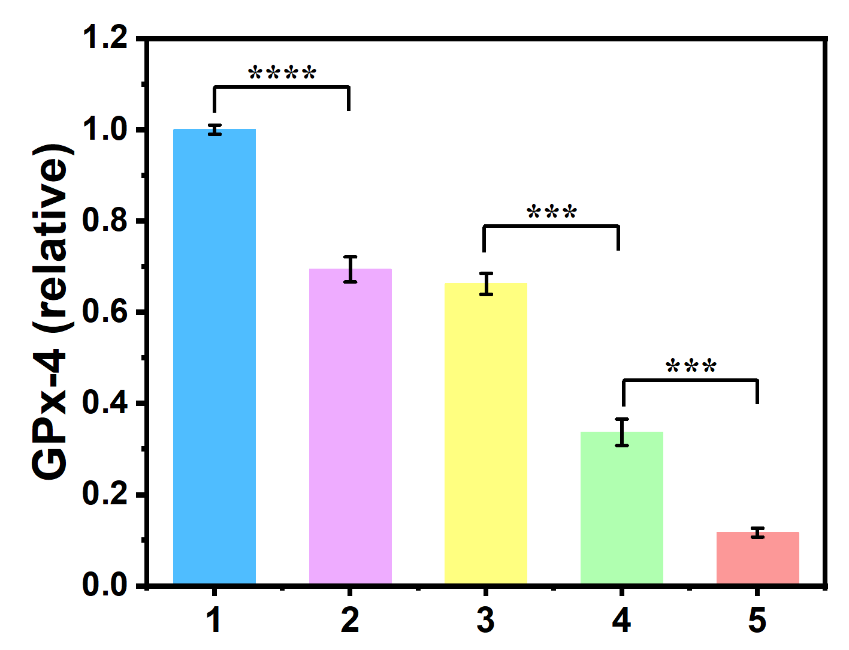


**Figure S20.** Quantification of the GPx-4 expression level in B16-F10 cells following different treatments in Figure 5g * represents P < 0.05, ** represents P < 0.01, *** represents P < 0.001, and **** represents P < 0.0001. The number of parallel detection n = 3.


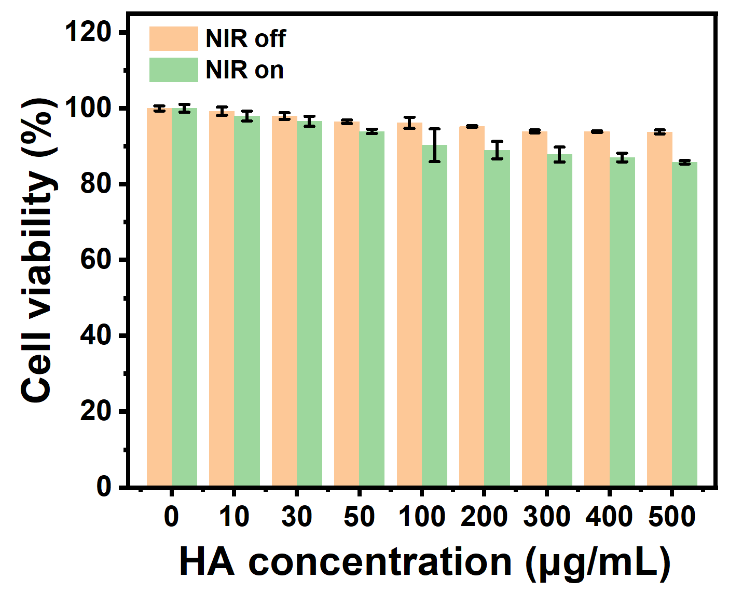


**Figure S21.** B16F10 cell viability after treatment with different concentrations of HA with NIR laser irradiation off and on (808 nm, 1.0 W/cm^2^).


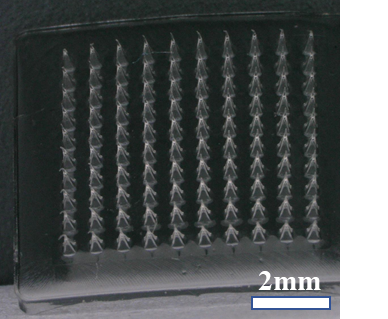


**Figure S22.** Typical bright-field microscopy image of HA MN patches.


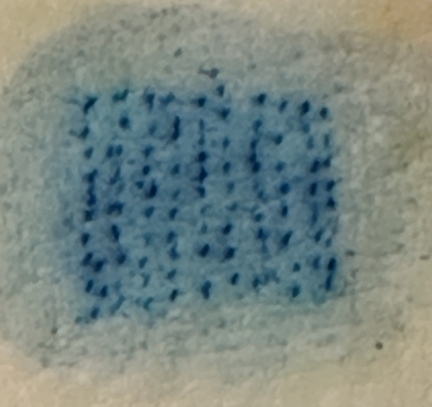


**Figure S23.** Typical photo of excised pig skin after methylene blue dye loaded MNs puncture.


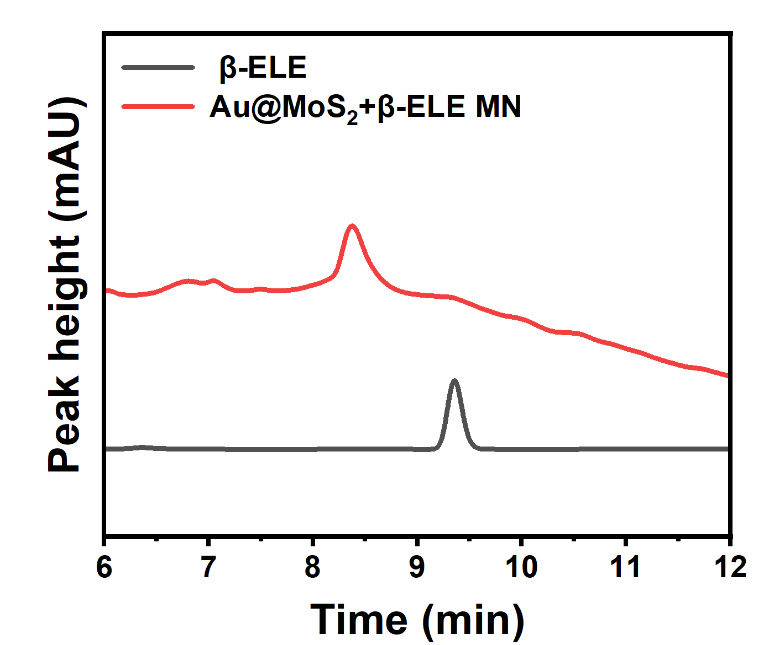


**Figure S24.** HPLC chromatogram of β-ELE in standard solution and release medium. The mobile phase was composed of water-acetonitrile (10:90). The flow rate was maintained at 1.0 mL/min, the column temperature was set at 40℃, and the injection volume was 20 μL.


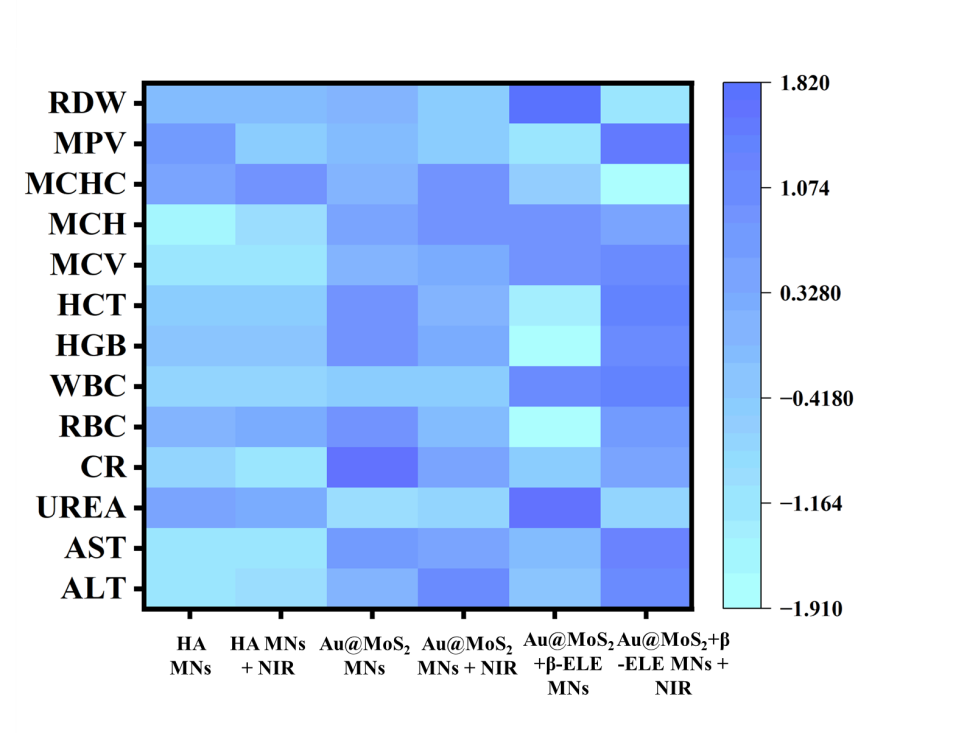


**Figure S25.** Heatmap for blood routine analysis of mice of different groups.

Table S1. Comparison of photothermal performance of different bimetallic plasmonic materials.

| Material | Photothermal conversion efficiency (%) | Ref. |
| --- | --- | --- |
| Pd/MoS_2_ | 43 | ^[4]^ |
| Au NPL@TiO_2_ | 42.05 | ^[5]^ |
| black phosphorus-Au | 36.1 | ^[6]^ |
| Bi_2_Se_3_-Au | 49.8 | ^[7]^ |
| NdVO_4_/Au | 32.15 | ^[8]^ |
| Cu_2_MoS_4_/Au | 34.69 | ^[9]^ |
| Ti_3_C_2_Tx-Au NPs | 43.4 | ^[10]^ |
| Au@MoS_2_ | 51.2 | This work |

Table S2. Comparison of K_m_ and V_max_ value of POD nanozymes

| Enzyme | K_m_, H_2_O_2_  (mM) | V_max_×10^-8^  （M S ^-1^） | BPNzyme  Yes (Y) or No (N) | Ref. |
| --- | --- | --- | --- | --- |
| La-RhRu | 0.72 | 23.4 | N | ^[11]^ |
| Fe_3_Ni-MOF | 0.1128 | 13.5 | N | ^[12]^ |
| Fe@MoS_2_ | 0.03 | 2.01 | N | ^[13]^ |
| Pt@Cu_2_O | 0.18 | 6.92 | Y | ^[14]^ |
| 3DRGO_Fe_3_O_4_-Pd | 0.02 | 5.58 | Y | ^[15]^ |
| Au@CeO_2_ | 0.007 | 0.82 | Y | ^[16]^ |
| MoS_2_ | 0.01299 | 3.40 | N | ^This work^ |
| Au@MoS_2_ | 0.00907 | 3.53 | Y | ^This work^ |

Table S3. Comparison of K_m_ and V_max_ value of GSHOx nanozymes

| Enzyme | K_m_, GSH  (mM) | V_max_×10^-8^  （M S ^-1^） | BPNzyme  Yes (Y) or No (N) | Ref. |
| --- | --- | --- | --- | --- |
| COFFePc | 9.78 | 81.7 | N | ^[17]^ |
| FeCo/Fe–Co | 1.775 | 107 | N | ^[18]^ |
| CDs@Pt | 1.04 | 746 | N | ^[19]^ |
| Cu_x_O@Bi_2_MoO_6_ | 0.606 | 14 | N | ^[20]^ |
| Bi_2_Fe_4_O_9_ | 1.85 | 92 | N | ^[21]^ |
| CuGQD/Pd@PSi | 0.4662 | 50.7 | Y | ^[22]^ |
| MoS_2_ | 0.21 | 18.6 | N | ^This work^ |
| Au@MoS_2_ | 0.05 | 38.2 | Y | ^This work^ |

**References**

[1] C. Jiao, W. Duan, X. Wu, Y. Shang, F. Zhang, M. Zhang, X. Chen, J. Zeng, C. Yang, *Analytical Chemistry* **2023**, *95* (30), 11316.

[2] Z. Tang, Y. Ju, X. Dai, N. Ni, Y. Liu, D. Zhang, H. Gao, H. Sun, J. Zhang, P. Gu, *Redox Biology* **2021**, *43*, 101971.

[3] J. Huang, G. Deng, S. Wang, T. Zhao, Q. Chen, Y. Yang, Y. Yang, J. Zhang, Y. Nan, Z. Liu, K. Cao, Q. Huang, K. Ai, *Advanced Science* **2023**, *10* (24), 2302208,.

[4] H. Lv, M. Lin, C. Yu, H. Wang, M. Li, L. Zhang, Z. Liu, Z. Chen, *Journal of Environmental Chemical Engineering* **2023**, *11* (5), 110375.

[5] F. Gao, G. He, H. Yin, J. Chen, Y. Liu, C. Lan, S. Zhang, B. Yang, *Nanoscale* **2019**, *11* (5), 2374.

[6] S. Li, Y. Zhang, W. Wen, W. Sheng, J. Wang, S. Wang, J. Wang, *Biosensors and Bioelectronics* **2019**, *133*, 223.

[7] X. D. Liu, B. Chen, G. G. Wang, S. Ma, L. Cheng, W. Liu, L. Zhou, Q. Q. Wang, *Advanced Functional Materials* **2021**, *31* (43), 2104424.

[8] M. Chang, M. Wang, M. Shu, Y. Zhao, B. Ding, S. Huang, Z. Hou, G. Han, J. Lin, *Acta biomaterialia* **2019**, *99*, 295.

[9] M. Chang, Z. Hou, M. Wang, M. Wang, P. Dang, J. Liu, M. Shu, B. Ding, A. A. Al Kheraif, C. Li, *Small* **2020**, *16* (14), 1907146.

[10] Z. Yu, L. Jiang, R. Liu, W. Zhao, Z. Yang, J. Zhang, S. Jin, *Chemical Engineering Journal* **2021**, *426*, 131914.

[11] J. Wu, Q. Liu, D. Jiao, B. Tian, Q. Wu, X. Chang, H. Chu, S. Jiang, Q. Yang, T. Liu, *Angewandte Chemie International Edition* **2024**, e202403203.

[12] Z. Mu, S. Wu, J. Guo, M. Zhao, Y. Wang, *ACS Sustainable Chemistry & Engineering* **2022**, *10* (9), 2984.

[13] S. R. Ali, M. De, *ACS Applied Materials & Interfaces* **2022**, *14* (38), 42940.

[14] J. Liu, Z. Zhang, J. Dong, A. Chen, J. Qiu, C. Li, *Talanta* **2024**, *266*, 125018.

[15] X. Zheng, Q. Zhu, H. Song, X. Zhao, T. Yi, H. Chen, X. Chen, *ACS Applied Materials & Interfaces* **2015**, *7* (6), 3480.

[16] C. Liu, M. Zhang, H. Geng, P. Zhang, Z. Zheng, Y. Zhou, W. He, *Applied Catalysis B: Environmental* **2021**, *295*, 120317.

[17] M. Rong, J. Liu, L. Lu, *Advanced Healthcare Materials* **2024**, 2400325.

[18] Y. Liu, R. Niu, R. Deng, S. Song, Y. Wang, H. Zhang, *Journal of the American Chemical Society* **2023**, *145* (16), 8965.

[19] L. Zhang, Q. Dong, Y. Hao, Z. Wang, W. Dong, Y. Liu, Y. Dong, H. Wu, S. Shuang, C. Dong, *Advanced Science* **2023**, *10* (30), 2302703.

[20] G. Zhang, S. He, J. Wei, P. Ran, H. Zheng, L. He, X. Li, *Small* **2025**, *21* (2), 2407281.

[21] Y. Zou, B. Jin, H. Li, X. Wu, Y. Liu, H. Zhao, D. Zhong, L. Wang, W. Chen, M. Wen, *ACS nano* **2022**, *16* (12), 21491.

[22] J. Zhao, W. Duan, X. Liu, F. Xi, J. Wu, *Advanced Functional Materials* **2023**, *33* (47), 2308183.
